# Evolution and Evaluation of Ultra-Low Temperature Freezers: A Comprehensive Literature Review

**DOI:** 10.3390/foods14132298

**Published:** 2025-06-28

**Authors:** Christos Kypraiou, Theodoros Varzakas

**Affiliations:** 1 Master’s Program in Quality and Technology Management, School of Science and Technology, Hellenic Open University, 26335 Patras, Greece; std160636@ac.eap.gr; 2 Department of Food Science and Technology, University of the Peloponnese, 24100 Kalamata, Greece

**Keywords:** ultra-low temperature (ULT) freezers, refrigeration technology, refrigerants, environmental sustainability, regulatory compliance, validation process, biological sample management, foods, healthcare, scientific research, energy efficiency

## Abstract

This review paper addresses the design and testing of ultra-low temperature (ULT) freezers, highlighting their critical functions in various industries, particularly foods, medicine, and research. ULT freezers operating at temperatures of −86 °C and lower have come a long way with improvements in freezing technology, for instance, from traditional vapor compression systems to new multi-stage refrigeration technologies. This progress has added operational reliability and energy efficiency, essential for preserving delicate samples and facilitating groundbreaking research. The article deeply explores the contribution of refrigerants to ULT freezer efficiency and sustainability. With the use of chlorofluorocarbons (CFCs), previously reliant on them, being prohibited due to environmental concerns, the sector opted for environmentally friendly substitutes like hydrofluorocarbons (HFCs), natural refrigerants, and hydrofluoroolefins (HFOs). Regulatory compliance is ensured by rigid validation protocols to guarantee ULT freezers are safe and meet quality requirements without compromising the integrity of the stored material. In addition to their wide-ranging advantages, ULT freezers also have disadvantages, such as energy efficiency, incorporating automation, the integration of IoT and AI for proactive maintenance, and the development of environmentally sustainable refrigerants. Adequate management strategies, including regular employee training and advanced monitoring systems, are vital to counteract threats from temperature variations and reduce long-term diminished performance. Finally, subsequent innovations in ULT freezer technology will not only aid in research and medical initiatives but also support sustainable practices, ensuring their core role as beacons of innovation in preserving the quality of precious biological materials and increasing public health gains.

## 1. Introduction

Industrial freezing systems and refrigerators play a critical role in various industries for which precise temperature management is essential [1,2]. Industrial freezers are classified based on their temperatures as deep-freezing, ultra-low temperature, and cryogenic [1,2,3,4,5]. Deep freezing freezers operate between −50 °C and −20 °C, ULT freezers between −86 °C and −50 °C, and cryogenic freezers below −100 °C [1,2,3,4,5]. This review focuses on ULT storage, which is crucial in pharmaceuticals, biomedicine, food storage, and research fields, protecting the integrity and efficacy of various products [1,2,5]. The need for ULT refrigeration has been more critical since 2020, following the emergence of the COVID-19 pandemic, which introduced new and complex requirements and demands for vaccines [3,4]. ULT temperature requirements require adopting more specialized solutions in the technology of refrigeration [5]. There is a variety of technologies available to meet modern demands, with the appropriate choice of the right technology being inextricably linked to the desired performance at temperature levels and particular operational requirements [6,7]. For this reason, this need has fostered very impressive advancements in refrigeration technology for achieving superior energy and thermal efficiency [8].

Traditional refrigeration systems, such as the vapor compression refrigeration (VCR) system, have long been the foundation of cooling technology [6,7]. These systems rely on cyclic compression, condensation, expansion, and evaporation of a refrigerant [6,7,9]. While effective for conventional refrigeration, VCR systems face significant challenges when attempting to achieve ultra-low temperatures due to the high compressor pressure ratios required [6,7]. For large-scale or bulk cooling applications, air refrigeration systems are often preferred. These systems offer higher temperature efficiency, stability, and uniformity, making them suitable for industrial-scale operations [9]. In contrast, for small-scale or laboratory applications, piston compressor-based systems are increasingly adopted due to their simplicity, affordability, and reliability [9,10,11,12].

Moving beyond traditional technologies, the development of modern ULT systems represents a significant advancement in refrigeration engineering. Modern systems are designed to achieve and maintain temperatures as low as −80 °C (±10 °C), with a strong focus on energy efficiency and temperature stability [13,14,15]. ULT freezers are critical in fields requiring precise thermal control, including biomedical research, stability testing, pharmaceutical storage, electronics, and advanced engineering [4,16,17]. Unlike conventional systems, ULT freezers often employ sophisticated multi-stage refrigeration methods, such as cascade refrigeration or free-piston engine technology [4,18,19,20]. These modern innovations are supported by improvements in materials, thermal insulation, and control systems, enabling greater energy savings and tighter temperature uniformity. Selecting the right ULT freezer requires evaluating key performance factors, including temperature control precision, power consumption, space constraints, and operational safety [4,18,19,20].

Given the increasing demand for regulatory compliance and validation in laboratory settings, organizations are now confronted with the challenge of choosing systems that not only fit their storage size but also meet demanding industry standards, recommendations, guidelines, and strict regulations [21,22,23]. The demand for ULT freezers in research and biobanking presents the necessity to ensure that such systems offer reliable performance and maintain sample integrity over long spans [24,25,26]. While ULT freezers offer tremendous advantages, such as maintaining sensitive samples and enabling path-breaking studies, they also bring along challenges that cannot be overlooked, such as energy consumption, maintenance requirements, power outages, robust monitoring and alarm systems, etc. [4,27,28].

This paper attempts to critically review the technological dynamics and development of ULT freezers by investigating several key research aspects focusing on their history on new technologies, innovation, and regulations. Firstly, it seeks to ascertain the developments in refrigeration technology that have governed the development of ULT freezers over the last few years, with a focus on transitioning from traditional systems to more complex methods that conform to greater levels of performance and energy efficiency. Secondly, the paper explores the impact of different refrigerants on the efficiency and environmental compatibility of ULT freezers, delving into the transition from ozone-depleting substances to eco-friendly alternatives. It also distinguishes that compliance must meet various industries, using the requirement of rigorous validation to ensure that one can ascertain safety and quality. Moreover, the study focuses on advanced ULT freezer technologies and a comparative analysis of those. Lastly, consideration includes the strengths and weaknesses of using ULT freezers in high-stakes applications, such as the medical and scientific research sector, where temperature stability is most crucial. In general, the scientific world has recognized the importance of ultra-low freezers, not only as storage means but also as a tool that maintains research and development [18,19,29].

With the right strategies, technologies, and processes, a sustainable future for storing sensitive samples that support advancement in science and healthcare can be created, resulting in a better and healthier society [18,19,30,31]. Innovation, participation, and sustainable performance in health call for continued investment in storage technology [4,19,29]. Collaboration, regulatory compliance, and ethics will enable progress toward a healthier human and sustainable science [19,29,30,31,32]. Moreover, the rapidly evolving conditions of refrigeration technology, driven by increasing environmental control, advances in technology, and the increased demand for ULT storage, underscore the urgent need for a thorough review of recent trends in ULT freezer systems [5,8,12]. With novel refrigerants, control systems, and advanced monitoring technologies emerging, there is a pressing need to close knowledge gaps, contrast the relative performance of new technologies, and establish best practices for implementation [3,5,23]. Systematic examination of the literature today is essential to synthesize these advances, inform industry standards, and inform future research agendas. Such a program ensures all concerned—researchers, industry professionals, and regulators—are offered the very best, evidence-informed results with which to make informed decisions, optimize operational performance, and meet demanding safety and sustainability criteria in ULT storage. It is, therefore, timely and imperative to facilitate such additional progress toward more efficient and eco-friendly refrigeration technology in high-risk scientific and medical uses. Thus, by investigating the abovementioned research aspects, this current review paper hopes to provide insight into the current status of ULT freezer technology and its significant developments in the field [4].

## 2. History

### 2.1. History of Refrigeration

Lab freezers are specialized equipment designed to meet the rigorous requirements of scientific and medical applications [33,34]. Even if they share the basic working principles of household freezers, by utilizing vaporized refrigerants, they are designed to yield much colder temperatures and with additional advanced features for high-accuracy temperature control, stability, and uniformity within the chamber [35]. Thus, this section focuses on the evolution of the ULT freezers.

The discovery of cooling dates back to the 18th century when Scottish physician William Cullen was able to create a vacuum at lower temperatures [35,36,37]. Vacuum refers to the pressure of gases in the sense that it describes space where atmospheric pressure is reduced or essentially non-existent [38,39,40,41]. When a vacuum is generated, temperatures can drop as the gases expand and pull heat out of the surrounding environment, and this is the foundation of the refrigeration processes [38,39,40,41]. Despite being primarily experimental, William Cullen’s 18th-century demonstration signaled the start of technological advancements that would later transform the industry in the 19th and early 20th centuries [35,36,38]. The commercial application of refrigeration by the late 1800s revolutionized the meatpacking, brewing, and food storage sectors, particularly in North America and Europe [35,36,38]. This invention made it possible to trade perishable goods over long distances and greatly improved food safety by laying the groundwork for global cold chains [35,36,38].

The application of refrigeration was first used practically in the 19th century through improvements like the vapor compression refrigeration cycle, which was invented by Jacob Perkins in 1834 and set the foundation for today’s refrigerators and freezers [35,36]. Jacob Perkins developed a process by which heat is drawn from a material or environment using vapors [35,36]. The cooling process of refrigeration includes numerous primary steps. To begin with, there is compression during which a compressor compresses a refrigerant vapor, increasing its temperature and pressure. Second, this hot pressurized vapor subsequently moves into the condenser, where it releases heat to the environment and condenses into liquid [35,36,37]. Third, the liquid refrigerant subsequently moves through an expansion valve, which causes a pressure reduction and consequent cooling of the refrigerant [35,36,37]. Finally, the cooled liquid enters the evaporator, where it absorbs heat from the environment, evaporates once more, and continues the cycle. This cyclical process forms the basis of refrigeration system functioning, allowing effective cooling [35,36,37]. The development of this technology has had a significant impact on the food sector, transportation, and storage commodities [16,39,40,41]. This system significantly improved food preservation and made it possible for year-round storage and transportation by the late 19th century, when it was widely used in sectors like brewing, meatpacking, and ice production [16,39,40,41]. Daily life and public health were improved by increasing industrial efficiency and laying the foundation for refrigeration [16,37,38,39,40,41].

During the beginning of the 20th century, advances in refrigeration technology continued to result in the emergence of mechanical refrigeration systems that were capable of achieving lower temperatures [16,37,38,39,40,41]. These machines were first used in commercial environments, such as meat processing factories and breweries [16,37,38,39,40,41]. The need for low-temperature preservation rose with the development of cryobiology at the midpoint of the 20th century when researchers looked for ways to freeze and preserve biological materials without loss of viability [35,37]. Bioscience required not only freezing but also conservation at very low temperatures to maintain cell structure and viability, and thus, the first ULT freezers were released [35,36,37,38,39]. These adaptations of standard freezers utilized more advanced insulating materials and powerful refrigeration units [35,42]. The initial commercially-produced −86 °C freezers entered the market during the 1960s by Thermo Fisher Scientific and Revco [35,36,43]. The development of ULT freezers in the 1940s and 1960s allowed for the long-term preservation of biological samples, tissues, and cells, which transformed bioscience and medical research [35,36,43]. The development of vaccines, reproductive medicine, and organ transplantation were all advanced by this invention [35,36,43]. The 1960s saw the commercial introduction of −86 °C freezers, which further increased pharmaceutical storage and biobanking and laid the groundwork for the current global biomedical infrastructure [35,36,43].

During the last few decades of the 20th century and early 21st century, monumental technological advancements improved the usability, reliability, and effectiveness of ULT freezers [28,34,35]. These developments have assisted in safely and efficiently storing valuable materials and samples [34,44]. Among the significant innovations was the insulation [45,46]. Over the decades, insulation products such as polyurethane have emerged as the norm, enhancing energy efficiency, efficient vacuuming, and temperature management [45,46]. The lack of frosting accumulation has also been a measurable advantage, reducing the maintenance frequency [45,46]. Additionally, the application of microprocessors in freezer systems during the 1980s brought a new era in the monitoring and control of temperature [35,36,47,48]. These sophisticated systems preserved samples in the required range of temperature and also had alarm functions for excursions into protective service of valuable materials [35,36,48]. The integration of microprocessors into freezer systems in the 1980s revolutionized temperature monitoring and control, greatly enhancing sample preservation accuracy [35,36,47,48]. These advanced systems enabled real-time temperature regulation and included alarm functions to promptly detect deviations, thereby protecting valuable biological materials from damage [35,36,47,48]. This innovation significantly increased the reliability and safety of storage in research and clinical settings [35,36,47,48].

With respect to energy efficiency, the demand for cleaner technologies has been motivating the development of energy-efficient ULT freezers during the 21st century [35,36,49]. Research into alternative refrigerants and more effective compressor technology has far reduced the environmental impact of such appliances [36,48,49]. Additionally, modern ULT freezers also come equipped with digital monitoring systems that provide real-time temperature control, along with internet remote access [48,50]. Technology is used to deliver maximum protection to precious samples such that there will be a speedy response to any complications [48,50]. For maximum ease of use, accessibility, and ergonomics, freezers have been made easy to use, maintain, and clean [48,50,51]. Features such as adjustable shelves and easy-to-read displays make them the ultimate user-friendly machines for maximum use in day-to-day practicality and convenience in their operations [28,52].

The development of ULT freezers is a testament to scientific and engineering advancements [35,37,53]. From the inception of refrigeration methods to recent advances in ULT technology, freezers have become vital devices for the maintenance of biological integrity and the success of scientific inquiry [35,37]. With further developments in technology, future ULT freezing has even more effective, sustainable, and inexpensive systems to further optimize their applications in research and industry [35]. Having in mind the above analysis, the development of laboratory freezer technology illustrates the intricate relationship between environmental responsibility, industrial demand, and scientific innovation. Early refrigeration pioneers like William Cullen’s vacuum cooling in the 18th century and Jacob Perkins’ vapor compression cycle in the 19th century established vital foundations that revolutionized industrial refrigeration and food preservation, but at first, they prioritized practicality over environmental impact [37,41,42,43]. Driven by the increasing demands of cryobiology and biomedical research, ULT freezers emerged in the mid-20th century, marking a significant shift that demonstrates how technological advancement closely tracks scientific demands [35,36,37,40]. Although energy consumption is still an issue, the late 20th-century integration of microprocessors and sophisticated insulation is another example of how small technological advancements have improved dependability, efficiency, and user safety [35,36,45,46,47,48]. It is clear that the development of lab freezers is not just a linear technological trajectory but rather a dynamic process influenced by broader ecological and societal imperatives. As the refrigeration industry looks to the future, striking a balance between cutting-edge performance and environmental stewardship is crucial [35,53,54,55].

### 2.2. History of Refrigerants

Energy efficiency, environmental sustainability, and regulatory compliance are all impacted by refrigerants, which are essential parts of cooling technologies. Selecting a refrigerant requires weighing advantages and disadvantages against new opportunities and possible risks, particularly in light of climate change and changing environmental laws. The past decades have seen the air conditioning and refrigeration industries facing a serious challenge of balancing performance with efficiency and environmental responsibility [55].

Chlorofluorocarbons (CFCs) were initially developed in the early 20th century and shortly became extremely popular as refrigerator propellants due to the fact that they were stable, non-flammable, and efficient in terms of energy [56,57,58]. With time, the refrigerants used in ULT freezers have gone through significant evolution due to environmental concerns and policy developments [50,51]. One of the most important evolutions is the phasing out of CFCs by hydrofluorocarbons (HFCs) as the major refrigerants [53,54,55]. This transition is one of the bigger changes in environmental policy and technological adaptation to reduce harm to the world [53,54,55]. Labs and a wide industry applied CFCs in the air-conditioning and refrigeration processes [56,57,58]. In the middle of the 20th century, however, scientific research indicated a serious environmental issue: CFCs were very destructive to the stratospheric ozone layer [54]. The ozone layer is critical in protecting life on Earth by degrading most of the sun’s deadly ultraviolet radiation [56,57]. Research in the late 1970s and 1980s showed that CFCs, once released into the atmosphere, would break down and release chlorine atoms [55,59,60]. These chlorine atoms subsequently caused the ozone layer depletion (ODP), creating primarily the annual Antarctic ozone hole [53,54,55]. More precisely, ODP is a number that measures how much harm a chemical compound can do to the ozone layer of the Earth compared to a reference gas, usually CFC-11, which is assigned an ODP of 1 [60,61]. The ozone layer is what protects us from damaging ultraviolet (UV) radiation from the sun [61,62,63]. High ODP chemicals are more harmful as they destroy ozone molecules in the atmosphere, which can lead to more UV radiation reaching the Earth, causing health and environmental problems [62,63,64,65,66].

More precisely, in response to the growing environmental risk caused by CFCs, countries from all around the world came together and signed the Montreal Protocol in 1987 [56,57]. The first-ever environmental treaty aimed at phasing out the use and manufacture of ozone-depleting substances like CFCs [53,54,57]. The protocol sets binding reduction targets on the consumption and production of CFCs, encouraging industry and researchers to identify safer substitutes [53,54,57]. With phase-out of CFC, hydrofluorocarbons were the major substitute [53,54,57]. HFCs are non-chlorine-containing and, therefore, do not deplete the ozone layer, which makes them a sought-after substitute [53,54,57]. Their chemical composition allowed efficient regulation of heat, which made them ideal for laboratory freezers that require precise control of temperature to preserve sensitive samples [53,54,57]. Although HFCs have overcome the problem of ozone depletion, new environmental problems have been created [53,57]. Innovation in the refrigeration sector was spurred by the 1987 signing of the Montreal Protocol, which signaled a shift in international environmental policy [53,54,57]. The switch from ozone-depleting chlorofluorocarbons (CFCs) to hydrofluorocarbons (HFCs) allowed refrigeration technologies to continue to be used, especially in laboratory and medical applications [53,54,57]. Despite being safe for the ozone layer, HFCs are strong greenhouse gases that contribute to global warming, so this change also brought about new climate problems [53,54,57].

HFCs are potent greenhouse gases that possess high global warming potential (GWP) [50,53,54,58]. Global warming potential, or GWP, is an index of the combined heat trapped by a specific greenhouse gas in the atmosphere over a certain time frame—usually 100 years—and relates it to the quantity of heat trapped by carbon dioxide (CO_2_), which has a GWP of 1. It is an index of how much warming a gas causes the planet compared to CO_2_. Therefore, efforts are on the rise to use low-GWP refrigerants like natural gases (e.g., CO_2_, ammonia) or newer synthetic substitutes (e.g., hydrofluoroolefins) in a bid to reduce greenhouse gas emissions and prevent global warming [50,58].

As much as climate change issues have been solidified, the environmental impacts of HFCs have fallen under control [53,54]. In turn, industries and governments are actively searching and investing in creating and using more environmentally friendly refrigerants with lower GWP but still maintaining the level of functionality needed for crucial laboratory and industrial uses [53,55,57]. The transition from CFCs to HFCs is an essential step in the development of cooling technology to address environmental needs [53,55]. In the future, studies will persist in the innovation of refrigerants that are efficient and environmentally sound [55]. Alternatives such as hydrofluoroolefins (HFOs), natural refrigerants such as ammonia or CO_2_, and emerging technologies remain in focus as sustainable substitutes for HFCs [53,55]. This significant advancement in cooling technology and the switch from HFCs to more ecologically friendly refrigerants strike a balance between industrial and laboratory performance requirements and environmental responsibility [53,55,57]. To lower GWP, governments and businesses are investing more in sustainable alternatives like CO_2_, ammonia, and hydrofluoroolefins (HFOs) [53,55,57]. The dedication to combating climate change while preserving critical refrigeration capabilities is demonstrated by this continuous innovation [53,55,57].

Overall, the history of refrigerants used in laboratory freezers is a mirror of the greater challenges and advancements of achieving a balance between technology needs and environmental management [37]. With increasing global priorities for sustainability, the refrigeration industry will continue to change, seeking refrigerants with minimal environmental impacts since they aid the essential roles of scientific research and medicine [35,37]. These developments draw attention to enduring problems, particularly the effects of refrigerants like CFCs and later HFCs on the environment, despite their usefulness, caused by ozone depletion and global warming [53,54,55,56,57]. Innovation toward more sustainable refrigerants was sparked by the global policy response, exemplified by the Montreal Protocol, highlighting the changing ethical and regulatory environment that increasingly influences technological advancement [53,54,55].

## 3. Refrigerants

Considering growing concerns over climate change and ozone depletion, there is a necessity to research various refrigerants, their characteristics, strengths, weaknesses, opportunities, and threats [60,62,63,64,65,66]. Thus, the current section strives to provide an overview of the main refrigerant classes and to evaluate their impact on sustainability and environmental safety [44,58,59,60]. A summary of the following analysis is presented in Table 1.

Hydrofluorocarbons (HFCs) have been widely used since the end of the 20th century and have high thermodynamic properties and broad applications, ranging from commercial refrigeration to air conditioning [58,66,67,68,69]. They have a critical negative characteristic, which is their high GWP [60,63]. R-134a (tetrafluoroethane) is one of the most widely used HFCs in refrigeration [58,66,67,68,69]. It is highly efficient and available with respect to energy and can be used for both low- and medium-temperature applications [58,66,67,68,69]. Despite this, its high GWP makes it not a desirable option to continue [58,66,67,68,69]. Moreover, R-404A is also a blend of HFC-125, HFC-143a, and HFC-134a [59,64,66,70,71,72]. This mixture is suitable for its cooling capacity, and while it is strong during low temperatures, it also possesses a high GWP [59,64,66,70,71,72]. The industry is gradually reducing the application of R-404A as efforts are made to eliminate high GWP refrigerants [59,64,66,70,71,72]. In addition, R-407C, made up of HFC-32, HFC-125, and HFC-134a, serves as a replacement for R-22 [59,66,73,74,75]. It possesses a lower GWP than some traditional HFCs but is still not the best environmentally, with its increased GWP over new alternatives [59,66,73,74,75]. Secondly, R-410A is another blend (HFC-32 and HFC-125), which is used for its high refrigerant charge and low noise [59,76,77]. As in the case of other HFCs, it is regulated under environmental laws based on its high GWP [59,76,77]. Finally, R-507A, a mixture of HFC-125 and HFC-143a, demonstrates good performance in low temperatures but suffers from the same environmental concerns regarding high GWP [62,63,65,66].

Hydrocarbons, including propane (R-290), isobutane (R-600a), and ethane (R-170), have been progressively utilized to replace HFCs since they are characterized by low GWP and high energy efficiency [67,71,78,79,80,81,82,83]. However, the flammability characteristics of hydrocarbons require strict safety measures when in storage and utilization [67,71,78,79,80,81,82,83]. While hydrocarbons are a promising avenue for green cooling technology, the hazards associated with them make stringent safety measures necessary [67,71,78,79,80,81,82,83]. R-290 (propane) is also lauded for having much less GWP than HFCs and has proven to be an acceptable substitute for use in refrigeration [67,71,78,79,80,81,82,83]. It has high energy efficiency as a significant advantage but, on the other hand, is flammable and poses problems that must be treated carefully [67,71,78,79,80,81,82,83]. R-600a (isobutane), meanwhile, is another eco-friendly option with low GWP. It has good performance and efficiency, but like R-290, it requires special safety measures since it is a flammable material [67,81,83,84,85]. In addition, R-170 (ethane) is described as having very low GWP and efficiency but a narrow range of application and flammability, thus limiting its use in practice [67,79,80,81]. Thus, because of their extremely low GWP and high energy efficiency, hydrocarbons offer a lot of potential [67,79,80,81]. Their application in residential and commercial refrigeration systems is growing, particularly in areas where safety precautions are sufficiently taken. However, because hydrocarbons are flammable, there are safety and regulatory issues, especially in areas with high population density [67,79,80,81]. Despite having favorable environmental profiles, this prevents them from being widely adopted [67,79,80,81].

Ammonia (R-717) is a high-performance refrigerant with a long history of use in large industrial refrigeration systems [80,86]. Ammonia (R-717) is a natural refrigerant that is appealing for large-scale refrigeration and food storage applications due to its high efficiency, low cost, and zero greenhouse gas footprint [80,86]. It is extremely effective in the cooling procedure and is capable of supplying much greater cooling capacity compared to most of its alternatives [80,86]. However, the toxicity of ammonia requires stringent safety standards [80,86]. Ammonia causes far less environmental harm compared to HFCs and is hence widely utilized for bulk uses such as food processing refrigeration and refrigeration plants [80,86]. However, due to its toxicity, ammonia systems should be well planned in design and operation to prevent any potential health risks [80,86]. Its toxicity has tight safety precautions while handling it, which necessitates the training of workers and facilities to fit in detection systems and safety locks to prevent accidents [80,86]. Thus, there are substantial regulatory burdens and risk management requirements, and despite its advantages, these risks prevent its widespread use, particularly in non-industrial sectors where safety infrastructure might be insufficient [80,86].

Carbon dioxide (R-744) is an outstanding refrigerant option with low GWP, non-flammability, and non-toxicity, and it is a natural refrigerant [80,87,88,89,90]. Heat pumps, mobile air conditioners, and supermarkets are using it more and more. Its physical characteristics, e.g., high operating pressure, require special equipment for safe use and efficient functioning [80,87,88,89,90]. CO_2_ is also increasingly being used in many applications, e.g., supermarket refrigeration, due to its favorable environmental balance [80,87,88,89,90]. Nevertheless, CO_2_ systems are also susceptible to the limitations of needing to employ high-technology equipment and additional up-front investment capital since the equipment used to work with high pressures is sophisticated [80,87,88,89,90]. Despite expanding market opportunities, this technical and financial barrier prevents widespread adoption [80,87,88,89,90].

Hydrofluoroolefins (HFOs) synthesized because of regulatory directions against high GWP molecules are a next-generation refrigerant that attempts to reconcile the best thermodynamic features of HFCs with extremely low GWP values [80,91,92,93,94,95,96,97,98,99,100]. HFOs display a promising move towards environmentally friendly refrigerants but are hampered by infrastructure readiness and the need for further studies on their long-term environmental implications [80,91,92,93,94,95,96,97,98,99,100]. R-1234yf (2,3,3,3-tetrafluoropropene) is the most recognized HFO, which has been approved for use in refrigeration systems in motor vehicles and for some commercial uses [80,91,92,93,94,95,96,97,98,99,100]. It possesses a GWP that is about 75% less than that of R-134a, and it is thus a superior environmentally friendly replacement [80,91,92,93,94,95,96,97,98,99,100]. Yet, some drawbacks come along with this refrigerant, such as its comparative novelty, which leads to a lack of availability and the necessity of new infrastructure and/or qualifications [80,91,92,93,94,95,96,97,98,99,100]. Moreover, R-1234ze (Trans-1,3,3,3-Tetrafluoropropene) is another low-GWP HFO applied in various refrigeration and air conditioning applications [80,94,95,96,97,98,99]. Similar to R-1234yf, it has faced regulatory scrutiny and is widely considered a replacement for higher GWP refrigerants [80,94,95,96,97,98,99]. Procurement and technology infrastructure constraints for both R-1234yf and R-1234ze are significant issues when firms transition to such newer pairings [80,94,95,96,97,98,99]. Thus, their adoption and expansion are threatened by their inadequate infrastructure, status as novel technologies, and unknown long-term environmental effects, such as the production of trifluoroacetic acid [80,94,95,96,97,98,99,100].

The need for environmentally friendly and energy-efficient refrigeration technologies is driving the increased adoption of natural refrigerants [80,81,83,84,85]. There is a major natural cooling tap, which is butane (R-600) [42,81,83,84,85]. R-600, also known as butane, is a natural refrigerant that has garnered attention due to its low global warming potential (GWP) [42,67,77]. Its capacity for good refrigeration makes it best suited for numerous applications, ranging from household freezers and fridges [42,67,77,80,81,83,84,85]. However, its flammability requires caution in its handling and storage [42,77,83,84,85]. This implies that facilities employing the use of butane will be required to adhere to certain safety requirements to reduce the chances of accidents [42,67,77,80,81]. The choice of the right refrigerant goes a long way to determining the efficiency and sustainability of the refrigeration system, and thus, the selection process becomes crucial for modern-day refrigeration industries and applications [42,77,80,81].

Apart from the primary refrigerant varieties, certain specialty blends and less common refrigerants have appeared in various applications [42,101,102]. These special cooling taps typically target specific performance characteristics or specific applications [42,101,102]. It is to be noted that continuous research to find effective refrigerants is not only aimed at finding those with low GWP but also at making them have adequate cooling performance and safety parameters required for various applications [42,101,102]. R-401A is a mixture of HFC-125, HFC-143a, and HCFC-22, employed primarily as a substitute for R-22 in some systems [42,101,102]. Although it is sufficient in its capabilities, its GWP rating is high, so it is viewed as a disruptor rather than something that will endure [42,101,102]. R-421A is an azeotropic blend that aims to replace R-22 with a reduced GWP [42,75]. There are still, however, supply issues, making its global acceptance and use in present installations difficult [105]. R-40 (Ethylene) and R-12 (Dichlorodifluoromethane) are among the phased-out refrigerants due to their toxic environmental impacts, such as ozone depletion and high GWP [106,107]. Nevertheless, in certain specialized applications, R-40 is recognized for its low GWP and non-toxicity but with restricted applications [103,104,105].

Hydrocarbons, ammonia, carbon dioxide, and HFOs are some of the pioneers in this transition, each of them having certain advantages and disadvantages [66,67,68,69,87,88,89,90]. Hydrocarbons are renowned for their environmental benefits but are tempered by safety concerns related to flammability [83,84,85]. Ammonia stands out as being both effective and affordable for industrial applications [80,86]. Toxicity creates threats that must be treated with the utmost caution and protective protocols [80,86]. HFOs are the new wave of innovation, coupling improved environmental standing with existing applications, but deployment may be delayed by infrastructure constraints and regulatory uncertainty [80,91,92,93,94,95,96,97,98,99,100]. Although, among the many different refrigerant options, carbon dioxide (R-744) stands out as one of the most promising options for sustainable refrigeration in the future [80,87,88,89,90]. Because of its extremely low global warming potential (GWP ≈ 1), non-toxicity, and non-flammability, R-744 is unique among natural refrigerants and addresses both safety and climate concerns [80,87,88,89,90]. CO_2_ is becoming more and more popular in applications like industrial freezers, heat pumps, and supermarket refrigeration because it does not deplete the ozone layer like many synthetic alternatives do [80,87,88,89,90]. Technological developments are making CO_2_ systems more dependable and accessible for commercial use despite their high operating pressure, which calls for specialized equipment and a larger initial capital investment [80,87,88,89,90]. Additionally, it can function well in low- and medium-temperature environments due to its thermodynamic efficiency, particularly in transcritical and cascade systems, which makes it a flexible option for a variety of industries [87,88,89,90,108]. R-744 is a leading contender in the global transition away from high-GWP refrigerants because it provides a balanced profile of performance, environmental safety, and long-term availability as the refrigeration industry shifts toward more sustainable practices [87,88,89,90,103].

The continuous innovation and adoption of alternative refrigerants rely not only on their environmental features but also on technological developments, policy regulations, and market readiness [87,88]. Efficient transition to such green alternatives is imperative in reducing the carbon footprint of the refrigeration and air conditioning industries and, in turn, contributing to greater environmental goals [42,98]. All steps taken in this transition must be backed by careful planning, investment in new technologies, and emphasis on safety such that the benefits of adopting new refrigerants are obtained without undesirable impacts [42,98]. The refrigeration industry stands today at a critical point in its history, and choices today will affect future generations and the well-being of our world [42,98]. The refrigeration industry is at a turning point in its history, juggling technological advancements with legal and environmental requirements. Because of their availability and performance, HFCs are still used, but their high GWP causes a shift toward low-GWP substitutes like hydrocarbons, ammonia, carbon dioxide, and HFOs. Every refrigerant group has distinct advantages and disadvantages, and adoption prospects are constrained by risks related to infrastructure preparedness, safety concerns, and regulatory pressure [102,109]. To facilitate a sustainable transition in refrigeration technologies, future developments should concentrate on improving safety procedures, growing infrastructure, and addressing environmental uncertainties.

## 4. Importance and Application of ULT Freezers

### 4.1. Importance of ULT Freezers

Storage at ultra-low temperatures is a crucial factor in pharmaceuticals, biomedicine, and research fields, protecting the integrity and efficacy of various products [1,32,110,111,112]. Since technology has progressed at a rapid rate, effective methods of storage, particularly sensitive materials such as pharmaceuticals, biological samples, and foods, have been in higher demand [1,111,113,114]. The success of modern medicine and food research also mostly depends on the ability to store sensitive samples in extreme conditions [115]. Not only do ULT freezers provide the assurance of safe and long-term storage, but they also provide the ability to develop new drugs, vaccines, and therapeutic protocols [116,117,118]. The progress of medical research, such as gene therapy and immunotherapy, requires stable and reliable storage of histological samples and biological materials to maintain their functionality [115,119].

Since global health crises are an ongoing reality, research and development will be required in order to prepare and effectively arm the medical community [115,116,119]. In addition, the expansion of vaccine and drug supply, particularly during global health crises, highlights the significance of ULT freezers [4,120,121]. Temperature control and storage conditions may result in the stability of vaccines, which has been well illustrated in the recent COVID-19 pandemic [3,4,122]. Clinical trials and documentation of results processes require stringent and rigorous storage protocols supported by the proper utilization of ULT freezers [120,121].

With evolving technology, the ULT freezer industry tends to implement innovation that reduces energy consumption and maximizes usability and security [115]. Advances in refrigeration technology, such as the use of more efficient refrigerants, integrated monitoring solutions with IoT (Internet of Things), and artificial intelligence solutions to forecast needs and control temperature, are most likely to have a gigantic impact on the handling of research and samples [123]. More precisely, ULT freezers’ operational management has been completely changed by the integration of AI and IoT technologies, which have resulted in notable gains in operational efficiency, data-driven decision-making, and dependability. IoT sensors built into these freezers send real-time data to centralized cloud-based platforms while continuously monitoring vital parameters like temperature, humidity, door status, and power supply [115,123]. Facility managers and researchers can maintain ideal conditions without having to be physically present thanks to this connectivity, which enables continuous monitoring of storage conditions from distant locations. This kind of real-time monitoring lowers the chance of sample loss or degradation by enabling the quick identification of any departures from predetermined parameters [123]. By evaluating the massive streams of data produced, AI algorithms further improve the performance of IoT-enabled ULT freezers. By detecting early warning indicators like irregular temperature fluctuations or sensor anomalies, advanced predictive analytics can predict possible equipment failures and enable preventive maintenance. AI could, for instance, suggest compressor maintenance before a breakdown happens, reducing downtime and guaranteeing continuous storage conditions [115,123]. AI-driven automation can also extend component lifespan, simplify maintenance schedules, and optimize refrigeration cycles for energy efficiency. Modern sample management is made possible by the combination of AI and IoT, which not only increases the safety and resilience of ultra-low temperature storage but also helps with cost savings and regulatory compliance [115,123].

Also, in a more globalized world, supply chain efficiency in delivering far-traveling samples requires safe and reliable cooling systems [47,115,123]. ULT freezers must be able to withstand several man-made and environmental factors at high speeds and in an efficient capacity so that there is a consistent and stable storage condition wherever these sensitive materials are being transported [115,116]. Additionally, government and global collaboration can also form a significant part of the creation of regulations surrounding the operation of ULT freezers [28,52]. Such collaboration can be in the form of setting common regulations or standards for sample storage and transportation [24,52]. The “World Health Initiatives” and the “World Health Organization” are some of the global organizations that can assist in cementing the regulatory process that will ensure the ongoing safety and integrity of samples across the world [18,124]. Moreover, data collection and analysis concerning the stability and efficacy of samples in ULT freezers will probably be among the most influential factors in the entire process of new therapeutic strategy development [24,52].

ULT freezers are governed by national and international healthcare policy [76]. Unlimited government agency funding for healthcare and research can enhance the security of samples and the preparedness of many industries to respond to abnormal and critical events [52,76]. Policy formulation for ensuring the promotion of research and regulation of ULT freezers is necessary in a bid to ensure the response to any emergency [115,123]. For example, in healthcare, ULT freezers are increasingly becoming the cornerstone of an expanded interrelation between technology, innovation, and health [115,123]. These products not only meet basic needs but are also central to new treatments and public health preservation [18,124]. Organizations are encouraged to estimate the value of this asset, both at the resources and at the research and development stage, in an attempt to enhance their ability to compete in a more changing environment [18,52,76,124].

### 4.2. Applications

ULT freezers are critical for the storage and handling of temperature-sensitive materials that require close stability for research and other scientific purposes related to medical applications [13,59]. Proper handling, temperature wide range, and use of these freezers are crucial for the success of a variety of samples, food, and research [13,82,109]. Users can set specific temperatures, ensuring these essential elements are kept within set parameters [59]. Moreover, to protect against unexpected temperature fluctuations from power loss or equipment malfunction, laboratory freezers in most laboratories have internal alarm systems and backup cooling systems based on CO_2_ or N_2_ [58,59,60]. These elements ensure continuous monitoring and automatic responses to protect stored material [58,59,60]. Explosion-proofed construction through the avoidance of combustible materials and locking devices can also contribute to security and safety [58,59,60,63,64]. Briefly, freezers in laboratories are precious equipment to the scientific world, providing invaluable support in the preservation of delicate materials [60,62,63,64,65,66]. Their advanced features, ability to operate under extreme temperatures, and protective measures make them considerably superior and more advanced compared to domestic models [60,63,64].

Microbiology is based on ULT freezers to store microbial cultures and other biological reagents [113,114,125]. Crops need to be stored at lower temperatures to maintain their viability and enable future experiments [112,125,126]. Biologists and microbiologists need their samples to be stored in a safe place so that they can maintain the crops’ vital biological characteristics, as well as their growth capability [112,126]. Moreover, ULT freezers make it possible for microbial cultures to be stored for long periods without loss of biological activity [112,125,126]. The ability to maintain crops under temperatures of zero by a margin provides the ability to conduct experiments with reliable results, which are crucial to the invention of new drugs, vaccines, and biotechnological products [112,114,126].

Apart from that, ULT freezers allow researchers to conserve microbial and plant species’ genetic diversity, thus making evolutionary biology and ecological research possible [113,114]. Such applications not only augment understanding of biological processes but also allow researchers to acquire essential evidence of biodiversity and natural resource conservation [113,125,126]. Additionally, pharmacogenetics and genetic research are also significant areas where ULT freezers are now a necessity [1,32,110,111]. Pharmacogenetics is especially relevant today as the process of tailoring treatments to fit unique genetic patient profiles is becoming more advanced [1,32,110,111]. ULT freezers create conditions for the continuous preservation of genetic material, which forms the basis of personalized medicine, wherein patients are treated more effectively and safely [1,32,110,111]. Freezing and genetic research are the foundation of discovering new treatments and understanding genetic elements that influence disease and health [1,32,110,111,127].

Genetic samples must be preserved to conduct experiments and obtain valid results [26,32]. These samples can be from studies, clinical trials, or biobanks and generally require storage conditions that ensure their full integrity [26,32,111]. Beyond storage, ULT freezers also help in analyzing such samples so that scientists can perform complex experimental procedures such as PCR (polymerase chain reaction), DNA sequencing, and transcriptome analysis [32,110,111]. This research furthers the understanding of the mechanisms of genetic diseases and drug resistance, facilitating the development of new medicines and therapies [32,110,111].

Pharmaceuticals, including small molecules and biologics, rely on precise storage environments [1,32,111]. Smaller molecular compounds are typically stored at temperatures ranging from −20 °C to −80 °C [1,32,111], where changes in temperature can lead to loss of activity or degradation. Organic substances like peptides and proteins often require even more critical conditions, approximately −80 °C to −20 °C, depending on their composition [111]. The class of large molecules, such as monoclonal antibodies, recombinant proteins, and viral vectors, are primarily stored at about −80 °C to −20 °C to maintain their structure and function [112,113,114,126]. Improper storage can compromise their efficacy, emphasizing the importance of optimal conditions [112,113,114].

Storage of biological samples, including DNA and RNA, must be maintained at temperatures from −196 °C to −80 °C to prevent decomposition [44,122,128,129,130]. The preservation of nucleic acids is most critical for accurate gene analysis, and proteins are kept between −80 °C and −20 °C to extend shelf life and maintain functionality [122,128,129,130,131]. Vaccines, especially mRNA vaccines, require storage at very low temperatures, such as −80 °C to −60 °C, due to their heat sensitivity, to ensure efficacy and avoid side effects [34,119,132]. Blood products, including blood and platelets, have specific storage needs, with blood preserved at −80 °C or lower for safety and platelets stored at 4 °C for short-term use or −80 °C for long-term [133,134,135,136,137].

Cell culture materials, such as cell lines and stem cells, are stored at cryogenic temperatures, typically between −196 °C and −80 °C, to keep viable cell architecture and biological function [126,138,139,140]. Tissue samples, including fresh, frozen, or paraffin-embedded, are stored within the same range to support histological analysis and diagnosis [141,142,143]. Preservation of genetic material (DNA plasmids, oligodynamic samples) must also be maintained at these ultra-low temperatures to prevent degradation and ensure experimental validity [144,145,146]. Such storage conditions are essential for research, clinical, and diagnostic purposes [147,148,149].

Regarding the food applications, storage of perishable foodstuffs such as frozen fruits, vegetables, meats, and dairy products is generally between −40 °C and −18 °C, with ultra-low temperature freezers capable of maintaining in the −40 °C range for long-term preservation of sensitive items like certain fruits and vegetables [12,35,41,48]. Proper temperature control in food storage is essential to slow microbial growth, reduce enzymatic activity, prolong shelf life, and preserve nutritional quality, flavor, and texture [12,41,48]. While everyday food storage is typically managed with regular freezers, the use of ultra-low freezers for food items is less common but crucial for specific high-sensitivity products or research purposes [12].

Storage at ultra-low temperatures is crucial for biotechnology applications, especially for preserving biological samples like microbial cultures, genetic materials, and cell lines [112,114,125]. ULT freezers enable long-term storage with minimal biological activity loss, supporting experiments that rely on maintaining sample viability and integrity [112,125,126]. They are vital for conserving microbial and plant species’ genetic diversity, aiding research in evolutionary biology and ecological conservation [113,114]. Pharmacogenetics and genetic research explicitly depend on long-term storage conditions that prevent the degradation of DNA, RNA, and other genetic materials, facilitating advances in personalized medicine and drug resistance studies [1,32,110,111].

Research involving genetic samples, including DNA plasmids and oligodynamic substances, requires temperatures between −196 °C and −80 °C to prevent decomposition and ensure accuracy in experimental results [144,145,146]. Environmental and clinical samples are also stored at −80 °C to preserve their biological and chemical stability, supporting reliable analyses in various scientific investigations [147,148,149]. To maintain their physical, chemical, or biological integrity, environmental samples—which fall under the category of research samples—need to be stored at extremely low temperatures, such as −80 °C. This temperature guarantees low microbial or enzymatic activity, preserving sample stability over time. Samples of soil, water, or air particles used in contamination studies or environmental monitoring are typical examples. Numerous studies [147,148,149] have confirmed the consistent storage at −80 °C, demonstrating its dependability across research fields. For environmental research findings to be accurate and reproducible, such strict requirements are essential.

The storage of these biological and genetic materials at ultra-low temperatures provides a foundation for numerous processes, such as PCR, DNA sequencing, and transcriptomics—everything from understanding genetic diseases to identifying new therapeutic targets [32,110,111]. The preservation of vaccines—including mRNA vaccines—necessitates storage at extremely low temperatures (−80 °C to −60 °C) to maintain their effectiveness and safety [34,119,132]. Blood products, tissue samples, and cell lines are similarly stored within the same temperature range to preserve cellular viability and prevent degradation, supporting ongoing research, diagnostics, and therapeutic applications [133,134,135,136,137,138,139,140,141,142,143]. In pharmaceutical research, storage environments carefully maintain small molecules, biological drugs, proteins, monoclonal antibodies, viral vectors, and other sensitive agents to avoid activity loss and ensure stability over time [113,125,126]. This precise control is essential for the development of new medicines, vaccines, and advanced therapies that rely on the integrity of biological samples and genetic materials stored in ultra-low temperature freezers.

As technological advances continue, improvement in storage devices and surveillance systems will be increasingly crucial across all industries, particularly in biotechnology and pharmaceuticals, which require high performance from products [120,147,148,149]. In total, beyond their technical function, the success concerning the integrity and security of samples stored in these freezers has additional implications [18,52,124]. ULT freezers not only store valuable samples but also make advancements in science possible, creating a bridge of mind between modern medicine and future discoveries that can change the trajectory of human medicine. Investment in ULT freezers is, therefore, a strategic choice with direct implications for the ability of the scientific community to generate decisions and innovations that will improve the quality of life and health of the population [115,123]. Continued growth and innovation in the field of such industries and research work may determine the future treatment and solutions that will address the healthcare problems of the future [18,52,123]. ULT freezers are not just storage equipment. They are beacons of innovation, lighting the way to breakthroughs that can save lives and transform the lives of tens of millions of individuals across the globe [18,52,115,123]. The importance attached to low-temperature technology reflects the close association between science and society, as scientific inquiry and development continue to impact public health and welfare [18,52,123].

Table 2 categorizes the above discussion of the products and their optimal storage temperatures, a factor that reflects their unique need for preservation.

## 5. Advanced ULT Freezer Technologies

### 5.1. Advantages and Challenges of ULT Freezers

ULT freezer usage is a protocol of advantages that make it an indispensable part of many scientific, industrial, and medical practices. However, their extensive use is not an easy ride. Below, we will explain in detail the advantages of employing ULT freezers. Although ULT freezers have many advantages, they also relate to problems that must be considered during their selection and use.

One of the most critical advantages of ULT freezers is that they are capable of delivering safe and long-term storage of sensitive materials [27,117,123,150]. ULT freezers provide temperatures typically between −86 °C and −40 °C, which are required to avoid biological change and combat any chemical reaction that can degrade the potency of stored materials [5,19,123,150,151]. The relevance of such effective preservation is justified by the growing need to manage delicate biological products, such as vaccines, genetic material, and biological drugs, which require specific storage requirements to maintain their quality [19,123,150,151,152]. ULT freezers protect these items from thermal deviations that would cause loss of integrity [19,123,150,151,152].

ULT freezers ensure a rigid and constant temperature, which is extremely crucial for product quality and safety [5,123,151]. Stability at temperature preserves drugs and samples under optimal conditions to prevent the danger of thermal oscillations that can lead to failure or degradation of the product [5,19,28,123,150,151]. Continuous temperature monitoring using in-built monitoring systems ensures that any change is detected instantly, allowing users to take action accordingly [52,151,153]. This feature reduces the danger and risk associated with possessing sensitive materials, especially in biomedical clinics and laboratories whose success rests on the integrity of their samples [123,143,150].

Temperature recording and monitoring in ULT freezers can be useful in providing product safety and quality data [5,47,123,150]. This capacity enables systematic research into storage conditions and modifications to the management process [19,50,123,150]. Such data can be determinative to make conclusions about product quality throughout storage and to make sustained improvements in storage processes [5,19,50,123,150]. In addition, the ability to store information as computerized documents allows for easier analysis and evaluation, thus averting problems potentially caused by human intervention errors [5,19,50,123,150,154]. This characteristic is particularly important where regulatory matters and approving processes are strict, such as in the pharmaceutical sector and biological research [50,123,150,154].

ULT freezers promote research and innovation in reproductive medicine and pharmacology [19,27,28,152]. With their ability for the preservation of fragile samples and biological samples, they help in the process of developing new drugs, vaccines, and biologics [5,19,150]. Maintaining the integrity of samples allows the researchers to perform experiments of high accuracy, and this enhances their ability to detect and verify new drugs of therapy [4,150]. To aid in egg and sperm preservation purposes, ULT freezers play a critical role in supporting procedures that allow the family to achieve their desired outcomes, offering other chances for infertile patients [4,5,19,52,150].

Phase change materials (PCMs) have been used more and more in the design of ULT storage equipment in order to enhance thermal performance, particularly in lowering temperature fluctuations during transient occurrences such as power interruption, door opening, and equipment failure [102,155,156]. PCMs operate on the principle of being capable of absorbing or releasing latent heat while undergoing their phase changes (most commonly from solid to liquid and vice versa), and in doing so, they can serve as thermal buffers in temperature-sensitive storage conditions [102,155,156]. PCMs can become the key to stabilizing temperature and protecting product safety. PCMs in such low temperatures most commonly take the form of specially engineered synthetic products or eutectic mixtures with precisely defined melting points coordinated with the working range of the freezer [102,155,156]. For example, in a ULT freezer, a PCM having its phase transition at −75 °C to −80 °C in storage compartments or wall panels could be utilized [102,155,156]. During a power shutdown or door opening of the freezer, the PCM would soak up the thermal energy, resulting in delayed temperature rise and providing corrective action with precious response time. From the point of view of energy efficiency, the usage of PCMs is able to reduce the rate of compressor cycling frequency in mechanical systems by reducing thermal load spikes, especially infrequent door openings or high ambient conditions [102,156]. This can extend the compressor life, reduce maintenance needs, and lower operating expenses. There are, nonetheless, some limitations and challenges: PCMs must be well selected so as not to cause thermal mismatch; their long-term stability as well as phase cycling longevity must be evaluated, and any leakage risk must be minimized by adequate encapsulation [102,155,156]. The use of PCMs in ultra-low freezers is an appealing option for enhancing system resilience, thermal buffering, and energy efficiency. Their ability depends heavily on integration method, material selection, and system compatibility with the remainder of the refrigeration apparatus [102,155,156]. Users and manufacturers of equipment ought to evaluate PCM performance through empirical study and provide this data within risk analysis and operational qualification procedures.

One of the most critical challenges of a ULT freezer is the massive consumption of energy to operate, and this can affect the cost as well as the environment [4,19,151]. They store extremely low temperatures for extended amounts of time, and this is what contributes to high electricity consumption [4,5,19,28,150,151]. Increased energy requirements also raise a second question regarding the environmental cost of ULT freezers, as an increasing rate of energy consumption has been linked with CO_2_ and other greenhouse gases [19,28,52,151,157]. Therefore, there is considerable need for the development of energy-efficient models and more generally sustainable alternatives that will reduce energy consumption, yet not at the expense of efficiency [19,28,52,151,157].

ULT freezers require periodic maintenance to ensure that they remain in optimal working condition [19,151]. Customers must follow strict maintenance schedules and carry out regular inspections to ensure that refrigeration units operate under normal conditions [19,28,52,150]. Any breakdown or default in maintenance can lead to serious consequences, such as the loss of valuable samples and products, which would have a serious impact on research budgets and timelines [28,52,150]. The reliability of ULT freezers is also important, considering that malfunctioning in refrigeration can compromise the security of stored materials [19,28,52,150]. That is why the users need to invest in monitoring systems and proactive measures that will ensure timely notification whenever there is any problem [19,150].

One other critical challenge of a ULT freezer is the problems caused by deviations in temperature [27,28,123]. If the temperature is interrupted, even for a short period, serious harm can be caused to stored products [27,28,123]. This situation can be caused by numerous reasons, including mechanical failures, human errors, loss of power supply, or even abrupt climatic upsets [27,28,123]. The damage caused by thermal fluctuation is typically irreparable for sensitive biological materials, such as tissues, vaccines, biological samples, or medications [4,27,28,123]. This means that loss of efficacy or degradation can have far-reaching consequences, both for patients and researchers [4,27,28,123,151]. Preparation degradation has the ability to lead to failures in experiments as well as cause losses of time and money [4,27,28,123]. This is why real-time temperature monitoring systems have to be created, and warning mechanisms have to be put in place [4,27,123,158,159]. The users must be capable of immediately responding to any interruption to prevent damage [4,27,28,123]. This plays a key role in maintaining the integrity of the hundreds or even thousands of samples that can be stored in a ULT freezer [4,27,159].

The future challenge for ULT freezers will be to invest in their sustainability and longevity to meet new demands and ever-evolving scientific demands [28,151]. The producers need to be constantly on the lookout for innovation and embrace new materials and technologies that will make these freezers compete not only with efficiency but also with energy efficiency [28,151,155,160,161]. The development of alternative strategies and efficient utilization of energy sources will be crucial to achieving top performance without paying too much [150,151,162,163]. Furthermore, manufacturers will also be required to partner with users in a quest to understand their needs in a bid to develop and design freezers that will keep up with the needs of today’s healthcare industry [5,150,151,163].

ULT freezers are an essential piece of equipment for modern scientific and industrial use, with significant advantages that improve the storage and handling of sensitive materials [4,123,151]. Their ability to offer secure and long-term storage, together with offering consistent temperatures, form the basis of many of the important processes in research and science [4,19,27,28,123,151]. Improving the quality of data and promoting innovation makes ULT freezers essential to scientific progress [27,28,151].

On the other hand, energy consumption issues, maintenance, and the need to maintain constant temperatures need to be faced and managed [4,19,27,28,151]. The users must be informed and trained about maintenance requirements and the potential effects of temperature disturbances [4,19,27,28]. With the present and future needs that are present in science, medicine, and industry, ULT freezers will need to adapt on a continuous basis [4,19,27,28,123,151]. Their advisement towards energy efficiency and greater reliability will be pivotal in making them valuable and sustainable in the long run [4,19,151].

### 5.2. Overview of ULT Freezers: Technologies, Configurations, and Energy Efficiency

ULT freezers are essential in a variety of industries, as explained above; thus, choosing the best ULT freezer requires knowing the variations in cooling techniques, system architecture, form factors, and specialized functionalities because there are many different technologies, configurations, and design elements available. The main differences between ULT freezers are examined in this section, and an overview of them is presented in Table 3, with an emphasis on how design decisions and technology advancements affect performance, energy efficiency, dependability, and fit for various storage requirements.

One of the main differences is the cooling technology used in ULT freezers. The most popular types of cascade refrigeration systems are the conventional ones, which use a cascade cycle with two hermetically sealed compressors operating in tandem [11,17,18,163]. These units are perfect for high-precision storage because they are dependable, tested, and can reach temperatures as low as −86 °C [11,17,18,79]. Stirling engine freezers, on the other hand, use helium as the working fluid and a free-piston design [164]. Because they do not use conventional compressors or oils, they are energy-efficient, environmentally benign, and require little maintenance [164].

The cooling capacity and dependability of a freezer are directly impacted by the compressor configuration [83,98]. For units that operate at higher temperatures—roughly −40 °C to −60 °C—or for portable and backup applications where simplicity and reduced costs are beneficial, single compressor systems are usually utilized [83,98]. For ultra-low temperatures close to −86 °C, dual compressor or cascade systems are the norm, offering dependable performance and balanced operation [83,98,132]. Multi-stage or multi-compressor configurations are useful for larger capacities or applications that require redundancy; they provide fail-safe operation and more cooling power [165].

ULT freezers’ usability and efficiency are also influenced by their form factor and design [4,8,83,98,164]. The most popular freezers in lab settings are upright models, which have capacities ranging from 300 to more than 800 L and provide easy access and movable shelves. Usually, they are employed for general storage in laboratories [4,8,84,98,164]. With capacities of up to 900 L, chest freezers offer excellent insulation and temperature stability, frequently at lower energy costs [4,98,164]. They are appropriate for long-term storage where temperature control is crucial due to their top-opening design [84,98,164]. Because they take up less room, under-counter models are perfect for point-of-care or clinical settings that need a moderate capacity [4,8,84,98,164]. Fieldwork and sample transfer are made easier by portable or mobile units, which are made for carrying biological samples. They can run on batteries or dry ice [4,8,84,98,164].

ULT freezers are further distinguished by unique features and systems tailored to particular applications. In order to minimize power consumption and lessen their impact on the environment, energy-efficient models use ENERGY STAR^®^ certified hydrocarbon refrigerants, such as R-290 and R-170 [51,56,98,165]. These systems are crucial in situations where continuous preservation is required. Furthermore, some freezers have modular or redundant compressor systems to guarantee optimal dependability during crucial processes like clinical trials or biobanking [51,56,98,165].

**Scheme 1:**
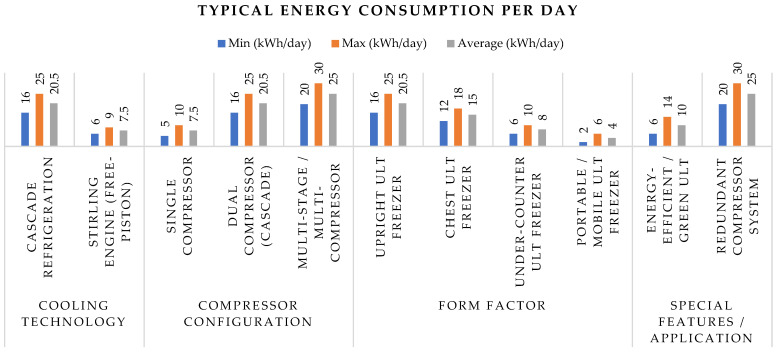
Typical energy consumption per day [4,8,18,51,56,79,164,165].

The energy consumption of ULT freezers varies greatly based on their form factor, compressor configuration, cooling technology, and unique features, as displayed in Scheme 1 [11,17,18,164,165]. Because of their intricate two-stage cooling cycles, traditional cascade refrigeration systems and dual compressor designs—which are frequently used for storage at temperatures between −80 °C and −86 °C—consume the most energy, averaging between 16 and 25 kWh per day [4,8,51,56,79,84]. The average daily consumption of multi-compressor systems used in high-capacity or redundant setups is even higher at 25 kWh. On the other hand, Stirling engine freezers, which use a free-piston system and helium as the working fluid, are among the most energy-efficient, using an average of only 7.5 kWh per day [17,18,98,164,165]. With fewer moving parts and smoother operation, linear compressor models provide a compromise at about 12.5 kWh/day [18,51,56,79,84]. Another important factor in energy efficiency is form factor [51,56,79,84]. While chest freezers are more efficient, with an average of 15 kWh/day due to better insulation and less air loss, upright ULT freezers match the high consumption of cascade systems. With daily averages of 8 and 4 kWh, respectively, under-counter and portable ULT freezers have the lowest consumption, making them perfect for small or portable applications [11,17,56,132,164]. Averaging 10 kWh per day, green ULT freezers balance sustainability and performance by using hydrocarbon refrigerants with low global warming potential. Ultimately, choosing a ULT freezer requires balancing trade-offs between operational dependability, energy efficiency, and storage capacity; therefore, energy performance is a critical component of long-term cost and environmental impact [18,79,164,165].

A number of factors, such as cooling technology, compressor configuration, design, and extra features catered to particular applications, must be carefully considered when selecting the best ULT freezer. The efficiency, dependability, and sustainability of these storage solutions have been greatly enhanced by developments in modular systems, thermal buffering with phase change materials, and environmentally friendly refrigerants. Organizations can maximize sample preservation, minimize operating expenses, and support long-term storage requirements by knowing the advantages and disadvantages of each system type. In the end, the best performance and protection for the most sensitive and valuable materials are guaranteed when technological capabilities are matched with operational objectives.

### 5.3. Industrial Examples of Advanced ULT Freezer Technologies

Refrigeration technology has progressed significantly in recent years, offering a variety of systems with different capabilities and features to meet the specialized needs of industry and science. Three prominent systems in refrigeration technology have been chosen to be researched and analyzed more in detail in this study and are the hermetic compressor cooling system (Barand: KW Apparecchi scientifici srl k66 hpl), the free-piston engine (Barand: Stirling su780xle), and the multi-compressor cooling system (Barand: klinge nmf-372). This analysis focuses on their different characteristics and capabilities.

**Figure 1:**
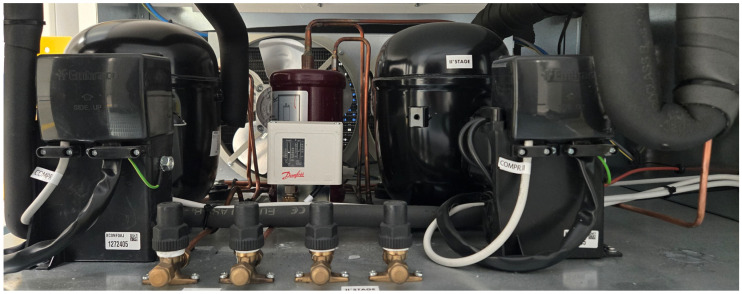
Hermetic compressor [Own Photograph by the authors].

Initially, the hermetic compressor cooling system uses hermetic compressors arranged in a vertical arrangement, as displayed in Figure 1 [166]. Hermetic compressors, fully closed, prevent liquid leakage, which makes this system ideal for maintaining high efficiency and reliability over time. The vertical arrangement is usually preferable in applications that require rapid temperature reduction or extremely low temperatures, as it allows for greater control and performance [156,166]. Also, the presence of a Miniature Circuit Breaker (MCB) identifier for protection ensures safety against electrical overloads, enhancing safety and operational reliability. The pressure gauge for monitoring condensation pressure (MR) helps diagnose system performance and ensures optimal operating conditions [156,166]. This cooling system is suitable for applications where precise temperature control is required, such as in scientific or industrial environments where environmental stability is crucial [156,166].

**Figure 2:**
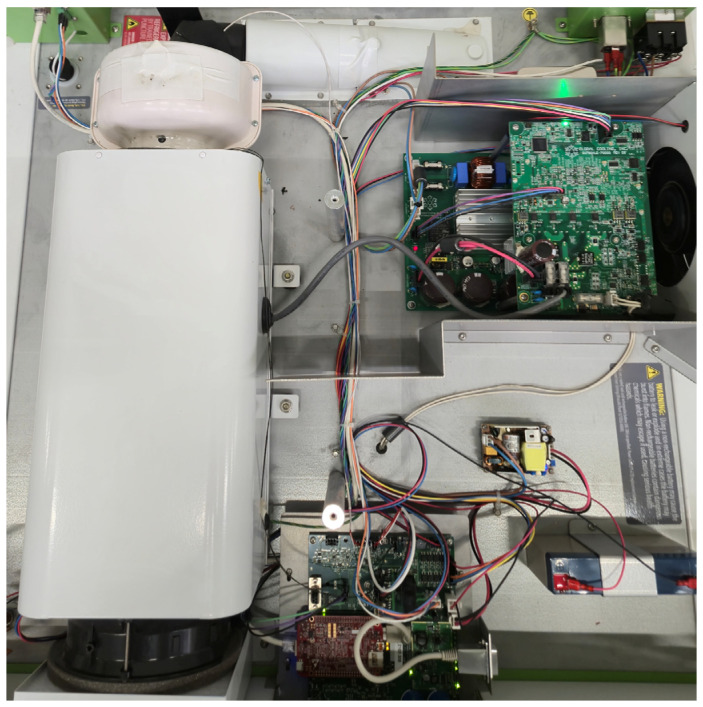
Free-piston engine [own photograph by the authors].

On the other hand, the free-piston machine uses technology that exploits helium as a working fluid, as displayed in Figure 2 [156,164]. These machines are known for their high efficiency and their ability to provide continuous temperature modulation, cycling on and off, which offers precise and smooth temperature control. Helium, as a working fluid, is efficient in heat transfer and allows operation at very low temperatures [15]. Continuous configuration capabilities allow the system to quickly adapt to changes in demand or thermal load while maintaining constant temperatures [156,164]. This feature makes the free-piston engine particularly useful in settings where temperature fluctuations can critically affect processes or stored materials. It is ideal for environments that require stable low-temperature conditions with minimal energy consumption and noise, such as medical or laboratory applications [156,164].

**Figure 3:**
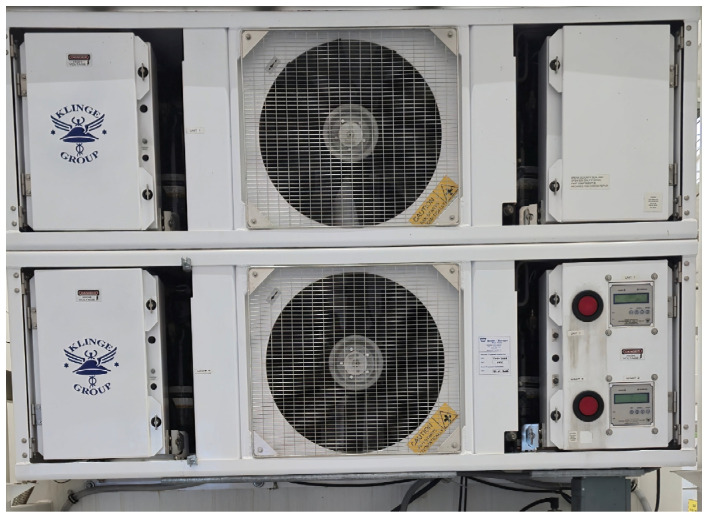
Multi-compressor [own photograph by the authors].

Finally, the multi-compressor cooling system has three different compressors, two for the high-temperature system using R134a refrigerant and one for the low-temperature system using R-23 refrigerant, as displayed in Figure 3 [156,165]. This dual system installation offers flexible temperature management in a wide range of conditions [156,165]. Using R134a for high-temperature modes and R-23 for low-temperature modes, the system is capable of effectively handling cooling variations [156,165]. The multi-compressor design optimizes performance in different temperature zones, enabling precision and efficiency in complex refrigeration applications [156,165]. This versatile system is particularly suitable for industrial applications, where different parts may require different temperature settings or where deep-freezing capabilities are needed [156,165].

ULT cooling technologies have advanced quickly, bringing with them a wide range of systems tailored to different industrial, preservation, and research requirements. The strengths and applicability of three well-known systems—the multi-compressor systems, the hermetic compressor, and the free-piston engine—are compared and examined below. Regarding cooling performance, energy efficiency, environmental impact, safety features, and connectivity, each system exhibits distinct capabilities that represent various priorities in various operating environments. To guarantee the integrity, sustainability, and safety of priceless biological samples and delicate materials, this analysis attempts to give a thorough overview that will direct the choice of the best cooling solution, customized to the unique needs of research institutions, industrial facilities, and transportation logistics as applicable.

All three systems offer excellent cooling performance, with the free-piston engine being recognized for its widest temperature range, giving it an advantage in storing various biological samples [156,164,165]. The hermetic compressor offers reliability through its sealed cooling system, as well as excellent thermal insulation, while the compressor system ensures reliable cooling with its dual system and its ability to operate in extreme conditions. Moreover, energy efficiency is critical for sustainability [156,164,165]. The free-piston engine consumes significantly less energy compared to traditional units and offers up to 40% energy savings. The hermetic compressor also has energy-saving programs, but it does not have the same scale as the free-piston engine [156,164,165]. The multi-compressor system, although it incorporates a dual system, also requires maintenance that can affect efficiency.

The hermetic compressor control system is highly advanced, with remote monitoring and data management capabilities; however, the free-piston machine similarly offers excellent temperature recording and monitoring through its parish interfaces. The multi-compressor system focuses more on cooling integrity in extreme environments and less on advanced management features [156,164,165]. Moreover, all systems either use natural refrigerants or contain features that reduce the environmental footprint [156,164,165]. The free-piston engine excels for its extremely low energy consumption and the use of environmentally friendly refrigerants, while the hermetic compressor offers energy programs and advanced insulation processes. The multi-compressor system uses refrigerants with minimal potential to deplete the ozone of the environment [156,164,165].

Safety is paramount for ULT systems, as minimizing risks is imperative when storing valuable biological samples [156,164,165]. The hermetic compressor has safety thermostats and optional voltage stabilizers, protecting the liner from voltage fluctuations. Correspondingly, the free-piston machine includes advanced security features, providing locks and PINs to access the GUI, ensuring that only authorized personnel can make changes [156,164,165]. The multi-compressor system also includes safety features through data control and temperature monitoring, reducing the possibility of load tampering [156,164,165].

Supporting functions are critical for decision-making in modern research facilities. All three systems offer advanced capabilities but with a different focused impact. The hermetic compressor provides the most sophisticated connectivity options, facilitating integration with laboratory networks and enabling real-time monitoring. The free-piston machine offers applications for monitoring in remote locations, with 40% energy savings. The multi-compressor system, although less focused on connectivity, offers excellent flexibility during transport and storage, with the possibility of automatic reverse operation containing safety features to protect the load.

During undercharging (loading products at higher temperatures), all three freezers experience a temporary thermal disruption from the entry of products at higher temperatures [156,164,165,166]. The hermetic compressor and multi-compressor systems recover faster compared to the free-piston engine system due to their high cooling capacity and extensive air circulation [156,164,165,166]. The free-piston system, while energy-conserving and steady-state stable, is slower to respond to sudden thermal loads due to its lesser power and single compressor cycle reliance. The multi-compressor setup has the benefit of redundancy, bringing in more cooling circuits as needed, making it capable of handling larger thermal loads with less temperature fluctuation—particularly important when storing large-volume or temperature-sensitive biologicals [156,164,165,166].

Door openings are a common cause of transient thermal disturbance. High-performance insulation and some form of temperature control are provided in all three systems to negate such disturbances. Reasonable thermal inertia is provided by the hermetic compressor system, together with stainless steel construction, but temperature spikes can occur if doors are left open for longer than a few minutes [156,164,165,166]. The free-piston engine, despite being efficiently engineered, may experience ±1 °C variability and may need recovery time, while its real-time GUI feedback via automated monitoring helps the operators to take prompt action to rectify the situation [156,164,165,166]. The multi-compressor system, due to its massive volume and dual cooling circuits, enjoys greater robustness with stable temperatures even during frequent or prolonged access and is suited for high-throughput labs [156,164,165,166].

On abrupt power failure, the response of every system varies in accordance with the insulation quality and system design. Free-piston systems are low in energy consumption and typically have battery-powered monitoring systems as a backup, but they possess low thermal mass and are thus more susceptible to temperature rise in case of disconnection from backup power [156,164,165,166]. Hermetic compressors have medium insulation and tend to be able to sustain subzero temperatures for several hours, provided that the door is not opened. Their functionality, however, is highly dependent on outside UPS or generator systems [156,164,165,166]. Multi-compressor design, often used in large repositories, incorporates greater insulation and may have thermal buffers or phase change materials to slow down internal warming. Plus, its system design permits staged restart upon return of power to minimize component stress [156,164,165,166].

Under transient conditions, the internal product temperature of products in storage is affected not only by the freezer’s capacity to stabilize air temperature but also by storage density and airflow patterns [156,164,165,166]. Hermetic compressor units possess medium air circulation, which may create localized hot or cold spots under transient conditions. Free-piston systems offer tighter temperature stability under normal operation but are less forgiving to heat ingress due to the low refrigerant mass and less aggressive cooling cycles [156,164,165,166]. The multi-compressor system, via its high volume and designed redundancy, is more effective in delivering homogeneous temperature distribution even under dynamic conditions [156,164,165,166]. When storing temperature-sensitive products like vaccines or cell lines, reliability and homogeneity under transient conditions are critical. Thus, technology choice needs to be made not only on the basis of steady-state operation but also on resilience to frequent operational disruptions [156,164,165,166].

At a time when research and science require strict storage standards, the three ULT systems analyzed represent market-leading choices. Each option seems suitable for different working and user conditions [156,164,165]. The final selection should be based on the specific needs of the research institutions, including external actors, infrastructure, and available resources, to ensure the integrity and sustainability of the stored samples. These technologies provide reliable and effective solutions, making a significant contribution to the advancement of science and industrial production, as well as to the protection of sensitive materials that require special storage conditions. The following Table 4 shows a comparative analysis of low-temperature cooling systems as derived from our market knowledge.

## 6. Regulatory Compliance

Evaluation of temperature uniformity and efficiency of refrigerating equipment is one of the core elements of quality assurance for many industries, including pharmaceutical, biological, and food industries [21,22]. Equipment for refrigeration, especially at extremely low temperatures, must be put through strict validation procedures in order to verify that it is in accordance with regulatory requirements and specifications for perishable storage [167,168]. Domestic refrigeration systems’ energy efficiency has been seriously impacted by Phase change materials (PCMs). Javeri-Shahreza et al. [169] used a model and incorporated a dynamic PCM component into the refrigerator model. Validation of the model came from comparison of the simulator to experimental data with and without PCM in the freezer cabinet. National and international policymaking can be a significant contributor to making the samples safe and propelling innovation in ULT freezers [21,22]. Regulators are required to communicate with industries and research organizations to establish regulations that will make the freezers qualify against stringent performance and temperature criteria [21,22]. Furthermore, financing the grant of money and research schemes is likely to catalyze studies into fresh and improved cold storage technology and consequently enhance innovative storage solution developments and deployment [21,22,167,168]. A summary overview of the regulations is presented in Table 5 below.

Management of operations and facilities for many industries is paramount to the efficiency and regulatory compliance of processes in line with regulatory standards [21,22,167,168]. Hence, storage of critical products and samples is necessary in various industries like biomedical research, healthcare, food storage, and manufacturing [21,22,166,167]. The functioning of ULT freezers plays a critical role in maintaining the integrity and lifespan of such products that must be kept constantly exposed to ultra-low temperatures in order to prevent deterioration and make them sustainable [21,22,166,167]. During ULT freezer testing, some key performance characteristics must be tested, such as temperature stability, temperature uniformity, peak fluctuation, recovery time, energy efficiency, operational noise, and general durability and reliability [21,22,167,168].

Temperature stability and uniformity are two critical elements for the evaluation of the performance. Temperature stability is the ability of a ULT freezer to retain a stable temperature over time with minor temperature variations [21,22,167,168]. This stability is necessary to prevent compromising sensitive products. In the pharmaceutical industry, for example, temperature instability can be detrimental to vaccines and biologics, rendering them ineffective [161,167,168]. High-quality ULT freezers must be designed to provide even low temperatures and, therefore, maintain a wide range of sensitive materials from temperature-induced degradation [21,22,166,167]. On the other hand, temperature uniformity ensures that the entire storage space within the ULT freezer remains at a constant temperature [21,22,86,167,168]. Effective insulation and well-designed airflow networks help to eliminate hot and cold spots inside the freezer’s chamber so that products can be stored anywhere without risk [19,151]. This is especially important in larger freezers, where sudden temperature gradients can be present from the center outward [19,151]. Impeccable temperature consistency must be ensured to provide equal environmental conditions to all samples and products, keeping the chances of local degradation to a bare minimum [19,151].

Peak variance is an essential performance metric that captures the largest deviation from the set temperature at any moment, including those infrequent but temporary fluctuations [19,20]. A ULT freezer is of the highest priority to those industries that have extremely sensitive materials, such as pharmaceutical and biotechnology industries, where slight variations in temperature can lead to reduced efficiency or alteration [19,20]. Laboratories whose equipment has to be validated with utmost accuracy are careful to install ULT freezers with stringent specifications to ensure maximum retention of their precious supplies [19,20].

Door opening recovery time is an important measurement, which determines the degree to which a freezer can recover to the specified temperature after opening [21,22,166,167]. Short recovery times are critical in applications involving fast access, such as research laboratories and pharmaceutical storage facilities [21,22,167,168]. Limiting exposure of sensitive materials to warmer air when opening doors prevents defrosting and degradation [21,22,166,167]. ULT freezers with rapid recovery facilities are particularly worth it in high-efficiency settings [21,22,167,168].

Energy efficiency is increasingly important because of the high energy costs associated with operating a series of ULT freezers [51,162]. The use of energy-efficient models can reduce operation costs and support organizational sustainability efforts [52,162]. ULT freezers that use natural refrigerants and variable-speed compressors can optimize cooling efficiency with less energy consumed [52]. The reverse is the case when it comes to older units that utilize conventional refrigerants and constant-speed compressors, which consume more energy and cost [52].

The performance and long-term reliability of ULT freezers are dependent on durability and the quality of construction [28,51,52]. The freezers must be built with high-grade materials that resist the toughness of continuous use [28,51,52]. Durable external frames and internally supported components guarantee the life of the units while cutting the risk of breakdowns [28,51,52]. Advanced alarm and monitoring systems that provide real-time performance data are also significant features, allowing users to easily address any issue that may undermine the integrity of their products stored [28,51,52].

ULT freezers are precious resources within many industries, particularly useful for the preservation of sensitive materials [22,161]. The freezers operate below temperatures much colder than −70 °C, allowing product integrity to be maintained, which otherwise rapidly degrades [21,22,135,170]. In view of the extreme importance of freezers in ULT, regulatory bodies such as the European Union (EU) and the United States Food and Drug Administration (FDA) have formulated comprehensive guidelines so that freezers in ULT are in line with stringent safety, quality, and efficacy standards [170,175]. This essay examines such regulatory contexts and the time-consuming process to confirm ULT freezers and provides some insights into how organizations apply such guidelines in day-to-day practice to make the world safe [170,171,175].

The European Union, as a result of its extensive regulatory standards, plays a significant role in deciding how materials are stored, like the ones that require ULT conditions. Firstly, Volume 4 of EudraLex clarifies the Good Manufacturing Practice (GMP) regulations regarding the production and storage of medicinal products [170]. These standards emphasize controlled storage conditions and arrange equipment such as ULT freezers to be certified so that they meet the required parameters [170]. GMP guidelines require stringent assessment of storage conditions so that there is no compromise on product quality [170]. Moreover, the GDP Guidelines (2015/C 95/01) focus on the supply chain dimension, highlighting that both storage and transport maintain product integrity from manufacturing through to delivery [171]. For ULT freezers, this means that the end-to-end supply chain can hold ultra-low temperatures without any deviation, avoiding potential spoilage of temperature-sensitive medicines [172]. Continuous monitoring and validation processes are required by these guidelines to maintain compliance [172]. In addition, Directive 2000/54/EC makes references to the safety of workers against the risks from exposure to biological agents, which can involve handling and storage in ULT freezers [173]. It demands adequate safety measures such that any staff members who work with these freezers are sufficiently trained and equipped [170,171,172,173,174]. Meanwhile, Directive 2009/41/EC legislates the use of genetically modified organisms (GMOs) with degrees of restriction in tandem with safe measures for storage, once again necessitating precise control of temperature and further facilitated through ULT freezers [173]. Finally, the European Medicines Agency (EMA) gives scientific advice that complements these guidelines by focusing on the quality of biological drugs stored under ULT conditions [174]. Although not enforceable by law, these recommendations have an impact on national requirements in all EU Member States and ensure a harmonized approach towards the validation of ULT storage [170,171,172,173].

The United States has a strong regulation by the FDA that secures storage requirements of products. Title 21 CFR Part 11 plays a key role in governing electronic records and signatures, data integrity, and traceability of temperature records of ULT freezers [175,176]. This section demands the inflexibility of the use of electronic monitoring systems with assurances that all data are secure and reliable. Sections 210 and 211 have cGMP regulations for drugs, demanding storage conditions, such as equipment like ULT freezers, to have strict operational requirements [175,176]. This ensures consistent production and storage conditions, ensuring product efficacy and patient safety [175,176]. The FDA’s Biotechnology Inspection Guide offers supporting materials and reports required to comprehend the intricacies of organic storage [175,176].

Apart from regulatory requirements, several industry standards offer additional guidance, guaranteeing holistic best practices in the validation of ULT freezers. Standards for (bio)processing equipment have been defined by the American Society of Mechanical Engineers (ASME) BPE-2014, which also affect the validation and design of ULT freezers [159]. These standards allow for an apparatus to ensure the performance and reliability of the equipment at extremely low temperatures. The Pharmaceutical Inspection Co-operation Program (PIC/S) has globally acknowledged recommendations (PE-009-9 and PE-009-11) to ensure Member States, storage sites, and manufacturing areas implement best-quality procedures [159].

The International Society of Pharmaceutical Engineering (ISPE) publishes significant guidelines, such as the Baseline Guide for Active Pharmaceutical Ingredients and the Good Practice Guide for Cold Chain Management, which reflect industry best practices for validating cold control systems in ULT freezers [21,22]. These reports emphasize the importance of chamber monitoring and mapping, promoting a scientific method to achieve temperature consistency in each storage chamber [21,22].

The European Commission revised the Good Distribution Practices (GDP) guidelines on 5 November 2013 (PIC/S), considering more advanced supply chains [170,171]. The storage needs of drugs must be complied with when stored and transported within the specified limits as proposed by manufacturers or on external packaging [171]. GDP emphasizes extreme vigilance for the integrity of the supply chain. The same is guaranteed through the preservation of drug quality from manufacturing to the final point of storage, distribution, and sale, ultimately ending at the end user or patient [171]. Storage and transport of pharmaceutical and biological material need to be monitored continuously with calibrated systems for continuous verification or qualified transport systems based on historical process data [21,22,171]. This includes the use of monitoring as a tool for continuous verification and validation of the process, along with defining facilities and equipment as part of standard daily inspection practices [21,22,171]. Apart from this, maintaining precise temperature control during the supply chain is difficult and an essential process in guaranteeing the quality of drugs, especially where precise temperature management matters [21,22,171]. Therefore, certification is a key quality assurance tool for the pharmaceutical sector, certifying that the equipment, facilities, and systems are working in the right way and are producing the intended results [21,22,171]. Storage facilities designation is a crucial and the most used criterion in worldwide standards [21,22,135,170,171].

It is essential to create national and international policies in order to ensure the security of samples and foster innovation in the creation of ULT freezers [21,22]. As the preservation of sensitive material in different fields must be strongly controlled, it is essential to perform collaborations between regulators, industry, and centers for research in order to guide the implementation of rules that respond to the current needs [21,22,170,171]. In particular, the FDA and the EU are critical in implementing and sustaining regulatory systems that ensure product quality and safety across all processes—storage, distribution, and production [21,22,170,171]. Compliance with the guidelines released by these organizations and the application of best practices in the handling of ULT freezers ensure uniformity and integrity of the samples [2,21,170,171]. Moreover, research grants funding and supporting new refrigeration technologies lead to continuous improvement in storage systems, which is crucial for the development of medicine and science [21,22,135,170,171].

In conclusion, the performance of the ultra-low temperature freezer is crucial for secure product storage of a broad scope of products across numerous industries [28,51,52]. Temperature stability, uniformity, peak fluctuation, recovery time from door opening, energy efficiency, operating noise, and durability are all matters of concern to watch out for when choosing a ULT freezer [51,52]. Through prioritizing such performance measures, organizations maintain the integrity of their critical samples and products and, ultimately, maintain operational success and protect public health [51,52]. Collectively, the teamwork of the regulators and industry stakeholders and the support of research are priceless in developing and implementing effective and secure storage solutions [21,22,167,168]. By reviewing regulatory processes and conformity, the call for the need to constantly educate and remain aware of controlled storage is strengthened, thereby creating a safer and more effective future in many different fields [21,22,167,168].

## 7. Conclusions

With all the above in mind, the history of ULT freezers has been significantly shaped by advances in refrigeration technology. There has been a transition, over time, from traditional vapor compression systems to more complicated multi-stage refrigeration systems [177]. Advances in materials and engineering have seen freezers featuring a low-temperature range of as low as −86 °C. Microprocessor integration applied in monitoring and temperature control has increased the reliability of operation, and better insulating materials, such as polyurethane, have improved the efficiency of energy. All these developments allow ULT freezers to provide constant temperatures, which is vital in the protection of sensitive biological materials and supporting state-of-the-art research in various fields [156,177].

Different models of refrigerants condition the effectiveness and eco-friendliness of ULT freezers. Previously, CFCs were widely used but phased out due to their destructive impact on the ozone layer [156]. HFCs were a temporary solution, giving excellent thermal performance but high GWP problems. The emphasis on sustainability today has led to the quest for replacements, such as HFOs, and natural refrigerants, such as ammonia and carbon dioxide, with low GWP values [156]. The selection of an appropriate refrigerant is important, as the same can directly influence both the performance of the freezer in terms of cooling, as well as its environmental impact, leading the way towards more eco-friendly refrigeration technology [156].

The advantages of ULT freezers are tremendous, particularly in applications critical to life, such as healthcare and scientific studies. Their ability to store sensitive materials like vaccines, biological specimens, and genetic material under secure, controlled, and long-term conditions preserves their integrity and functionality [156]. Further, ULT freezers enable scientists to plan experiments and trials with exact accuracy, enabling enhancement in biotechnology, pharmacology, and reproductive medicine [156]. But there are also problems, such as high energy consumption and maintenance, which can lead to operational costs and certain risks from temperature fluctuations. Problems must be aggressively monitored and proactively managed to overcome risk and achieve the best performance [156].

ULT freezer compliance specifications are needed to ensure the safe storage of sensitive materials in a wide variety of industries. Regulatory agencies, like FDA and EMA, have promulgated good guidelines to manage the operation of such freezers, including checking equipment certification, as well as stern validation protocols. In ensuring an efficient long lifespan, along with the competence of ULT freezers, as per regulatory policies, stakeholders must spend on certain strategies and good practices [156]. Comprehensive and ongoing training of staff in operation, maintenance, and the importance of temperature control is essential in quality control maintenance [156]. The application of sophisticated monitoring systems that issue real-time warnings makes it easier to quickly identify and correct any temperature deviations or mechanical failures, thereby protecting valuable samples. Users and manufacturers must also collaborate to adopt energy-saving technologies like improved refrigerants and enhanced insulation [177]. By prioritizing routine maintenance and having well-defined procedures, stakeholders will be able to reap the maximum amount of operating time and efficiency from ULT freezers, maintaining compliance with industry standards and protecting public confidence in stored biological material [156].

Environmental concerns play an increasing role in the choice of cooling technologies. HFC-based refrigerants used in hermetic and multi-compression systems are being phased out due to the high global warming potential [54,57]. The free-piston engine offers a viable alternative by allowing the use of natural refrigerants such as helium and nitrogen, which align with current regulatory trends favoring low GWP solutions [53,55]. However, despite their environmental benefits, free-piston engines require specialized maintenance and know-how, limiting their widespread adoption in commercial ULT applications [35]. Multi-compressor systems, while still relying on conventional refrigerants, can be optimized with alternative low-GWP refrigerants, such as HFOs or CO_2,_ to reduce environmental impact [60,88].

The study’s findings provide useful insights into industries that rely on ULT freezers, including biomedical research, pharmaceuticals, and biotechnology [177]. The superior temperature uniformity of multi-compressor systems makes them ideal for large-scale bio-storage and clinical specimen storage. However, higher energy consumption requires strong energy management strategies to compensate for operating costs [27]. Free-piston engines, with their energy efficiency and environmental advantages, are suitable for applications where sustainability and long-term cost savings are prioritized. However, their slow recovery times limit their suitability for applications that require frequent access or high thermal resistance [4]. Hermetic compressors, despite their lower efficiency, remain viable for budget-conscious applications where moderate temperature fluctuations are acceptable [44].

The future of ultra-low temperature freezers is extremely bright, not only for supporting research and medicine but also for supporting environmentally friendly practices [156]. The future growth of these technologies will be influenced by cross-disciplinary cooperative work among industry professionals, scientists, and regulatory bodies to ensure that ULT freezers are maintained as central tools of today’s scientific exploration [156]. By their focus on the invention and application of effective refrigeration technologies, the industry will work toward fostering a culture of accountability, safety, and innovation that will result in innovations capable of improving public health as well as advancing the cause of many different sciences [156].

Future research should investigate long-term operational stability, maintenance costs, and lifecycle environmental impacts in different refrigeration systems [50]. In addition, advances in alternative refrigerants and hybrid refrigeration technologies could provide innovative solutions to balance energy efficiency, environmental sustainability, and reliability of performance [62]. Future studies should explore the integration of phase change materials (PCMs) and AI-driven adaptive cooling control to enhance temperature stability while minimizing energy consumption achieving validation [169]. Studies could delve into longitudinal analyses of ULT freezer performance over extended periods, looking at how advances in technology affect operational efficiency. In addition, investigating user behaviors regarding freezer use and maintenance practices can yield actionable insights that align operational practices with technological capabilities. In addition, expanding this research to include a wider variety of environments and use cases can help identify industry-wide best practices that offer significant energy savings while ensuring product safety and service life. Researchers can also consider the possibility of integrating smart technology into freezer systems to provide real-time monitoring, predictive maintenance alerts, and automated operational adjustments based on environmental conditions [156]. Exploring the potential benefits of demand response strategies to freezer operations could yield insights into how to manage energy consumption during peak hours while maintaining efficiency [153]. Combining these strategies with evolving machine learning technologies can inform user-generated practices that lead to improved user experience and operational efficiency [156].

In conclusion, this review recognizes the significant advancement of ULT freezers and their inherent use in preserving sensitive materials and facilitating critical research in biotechnology and medicine. The analysis of other forms of refrigerants proves the trend in the future to be ecologically friendly decisions, while conformity requirements analysis and testing procedures identify the need to honor stringent safety controls. The review also describes the advantages and disadvantages of installing and operating ULT freezers, providing great insights into best practices and management. In addressing these topics, this review contributes to the development of the subject area through the provision of a comprehensive understanding of the technological and regulatory context of ULT freezers. However, note that there are areas of opportunity, notably in energy efficiency and the use of new monitoring technologies, that are ripe for additional research. Continued study in these areas not only stands to improve the effectiveness and sustainability of ULT freezers but will also keep them at the forefront as tools for driving scientific progress and improving public health outcomes.

## Figures and Tables

**Table 1:** SWOT analysis of refrigerants.

Group	Type	Attribute	Strengths	Weaknesses	Opportunities	Threats	References
Hydrofluorocarbons (HFCs)	R-134a	Tetrafluoroethane	Good energy efficiency, widely available	High GWP, climate impact	Transitional use in systems already reliant on HFCs	Global HFC phase-down, regulatory pressure	[58,66,67,68,69]
	R-404A	HFC-125, HFC-143a, and HFC-134a blend	Strong low-temperature performance	Very high GWP	Use in existing industrial setups	Obsolescence due to environmental regulations	[59,64,66,70,71,72]
	R-407C	HFC-32, HFC-125, and HFC-134a blend	Replacement for R-22, lower GWP than R-22	Still relatively high GWP	Retrofit applications	Market shift to lower-GWP alternatives	[59,66,73,74,75]
	R-410A	HFC-32 and HFC-125 blend	High capacity, quiet operation	High GWP, facing phase-out	Short-term high-performance use	Regulatory bans and alternatives like HFOs	[59,76,77]
	R-507A	HFC-125 and HFC-143a mixture	Stable performance at low temperatures	High GWP	Specialized industrial systems	Environmental and legislative pressure	[62,63,65,66]
Hydrocarbons	R-290	Propane	Very low GWP, high efficiency	Flammable	Domestic/commercial systems with proper safety	Safety regulations in populated areas	[67,71,78,79,80,81,82,83]
	R-600a	Isobutane	Low GWP, good performance	Flammable	Home refrigeration, small systems	Public perception, installation restrictions	[67,77,80,81,83,84,85]
	R-170	Ethane	Extremely low GWP, high efficiency	Flammable, limited use	Niche cryogenic applications	Narrow market scope due to safety limits	[67,79,80,81]
Ammonia	R-717	Ammonia	High efficiency, low cost, No GWP	Toxicity concerns	Large-scale refrigeration, food storage	Risk of leaks, regulatory burden	[80,86]
Carbon Dioxide	R-744	Carbon dioxide	Low GWP, non-toxic, non-flammable	High pressure requirements	Supermarkets, heat pumps, mobile A/C	Cost of high-pressure systems	[80,87,88,89,90]
Hydrofluoroolefins (HFOs)	R-1234yf	2,3,3,3-Tetrafluoropropene	Ultra-low GWP	Limited infrastructure, new tech	Automotive A/C, OEM adoption	Environmental uncertainty (e.g., TFA formation)	[80,91,92,93,94,95,96,97,98,99,100]
	R-1234ze	Dia-1,3,3,3-tetrafluoropropene	Ultra-low GWP	Infrastructure not yet mature	Stationary air conditioning	Uncertain long-term performance data	[80,94,95,96,97,98,99]
Natural Cooling Taps	R-600	Butane	Low GWP, effective cooling	Flammable	Household refrigerators	Safety standards and market limitations	[42,67,77,80,81,83,84,85]
Special Cooling Taps	R-401A	HFC-125, HFC-143a, and HCFC-22 blend	R-22 replacement	High GWP, transitional	Temporary retrofit use	Not sustainable long-term	[42,101,102]
	R-421A	Azeotropic mixture to replace R-22	Lower GWP than R-22	Limited market availability	Retrofit for older R-22 systems	Competing low-GWP solutions becoming standard	[42,75]
Inert Gas Refrigerant Taps	R-40	Ethylene	Low GWP, non-toxic	Very limited applications	Experimental or niche systems	Minimal commercial demand	[103,104,105]
Other Cooling Taps	R-12	Dichlorodifluoromethane—has been withdrawn due to ozone depletion	Good low-temp performance	Ozone depletion, high GWP	Only in legacy systems	Banned, legally restricted	[106,107]
	R-22	Chlorodifluoromethane—withdrawn due to HFC regulations	Good efficiency, historic widespread use	High GWP, phased out	Retrofitting with replacements	Global withdrawal under Montreal Protocol	[73,74,75]

**Table 2:** Product items and storage.

Category	Product Type	Storage Temperature	Examples	References
Pharmaceuticals	-Small molecular drugs	−80 °C to −20 °C	Acetaminophen, ibuprofen, aspirin	[1,32,111]
	-Organic (e.g., peptides)	−80 °C to −20 °C	Insulin, glucagon, vasopressin	[1,32,111]
Large Molecular/Biological	-Monoclonal antibodies	−80 °C to −20 °C	Adalimumab (Humira), trastuzumab (Herceptin)	[113,125,126]
	-Recombinant proteins	−80 °C to −20 °C	Recombinant human growth hormone (rhGH), erythropoietin	[112,113,114,125]
	-Therapeutic proteins	−80 °C	Interferon-beta, tissue plasminogen activator (tPA)	[112,113,114,125]
	-Enzymes	−80 °C to −20 °C	DNA polymerase, reverse transcriptase, trypsin	[12,35,41,48]
	-Viruses	−80 °C or −196 °C	Adenovirus vectors, influenza virus stocks, lentivirus	[10,64]
	-Antibodies against RNA lines	−80 °C to −20 °C	Anti-miRNA antibodies, anti-snoRNA antibodies	[44,122,131]
Biological Samples	-DNA/RNA	−196 °C to −80 °C	Genomic DNA from blood, total RNA from tissues	[144,145,146]
	-Proteins	−80 °C to −20 °C	Purified histones, protein lysates from cells	[64]
Vaccines	-mRNA vaccines	−80 °C to −60 °C	Pfizer-BioNTech (BNT162b2), Moderna COVID-19 vaccine	[34,119,132]
Blood Products	-Blood	−80 °C or cooling (<4 °C)	Whole blood units, red blood cell concentrates	[2,133,134,135,136,137]
	-Platelets	4 °C for short-term storage; −80 °C for long-term storage	Apheresis platelets, pooled platelet concentrates	[2,133,134,135,136,137]
Cell Culture	-Cell lines	−196 °C to −80 °C	HEK293, CHO cells, HeLa cells	[126,138,139,140]
	-Fetal/stem cells	−196 °C to −80 °C	Human embryonic stem cells, mesenchymal stem cells	[126,138,139,140]
Tissue Samples	-Fresh frozen samples	−196 °C to −80 °C	Frozen liver biopsy, tumor tissue slices	[142,143]
	-Paraffin (formalin) samples	−80 °C to −20 °C (for long-term storage)	FFPE tumor sections, preserved kidney samples	[142,143]
Genetic Material	-DNA plasmid	−80 °C	Plasmids used for gene expression, cloning vectors	[144,145,146]
	-Oligodynamic	−80 °C to −20 °C	siRNA oligos, antisense oligonucleotides	[144,145,146]
Research Samples	-Environment samples	−80 °C	Air particulate filters, contaminated soil extracts	[147,148,149]
	-Clinical samples	−80 °C	Nasopharyngeal swabs, serum from patients	[147,148,149]
	-Experimental observations	−196 °C to −80 °C	Cryopreserved test specimens, bioassay controls	[147,148,149]
Food (Perishables)	-Frozen fruits and vegetables	−40 °C to −20 °C (for long-term storage; in ultra-low freezers)	Frozen strawberries, green beans, corn	[12,35,41,48]
	-Meat and seafood (frozen)	−30 °C to −18 °C	Frozen beef cuts, salmon fillets	[12,35,41,48]
	-Dairy products (frozen)	−30 °C to −20 °C	Frozen cheese, ice cream, butter blocks	[12,35,41,48]
	-Ready-to-eat frozen meals	−30 °C to −18 °C	Frozen lasagna, chicken stir-fry packs	[12,35,41,48]

**Table 3:** Overview of ULT freezers.

Category	Type	Specifications	References
1. Based on Cooling Technology	Cascade Refrigeration System (Traditional)	Most common ULT freezer technology. Uses two hermetically sealed compressors and refrigerants in a cascade cycle. Can reach temperatures of –80 °C to –86 °C. Pros: Proven, reliable. Cons: Higher energy use, more moving parts. Energy Consumption: 16–25 kWh/day. Examples: Thermo Fisher TSX, PHCbi MDF series, Haier DW-86 series.	[11,17,18,79]
	Stirling Engine (Free-Piston)	Uses a free-piston Stirling engine with no oil or traditional compressor. Environmentally friendly (helium gas as the working fluid). Ideal for energy efficiency and low maintenance. Energy Consumption: 6–9 kWh/day. Examples: Stirling Ultracold SU780XLE.	[164]
2. Based on Compressor Configuration	Single Compressor	Not typical for –86 °C but used in –40 °C to –60 °C units. Simpler and cheaper design, often used for transport or backup. Energy Consumption: 5–10 kWh/day.	[83,98]
	Dual Compressor (Cascade)	Two-stage cascade system, standard for most –80 °C to –86 °C ULT freezers. Reliable performance with moderate energy consumption. Energy Consumption: 16–25 kWh/day.	[83,98,132]
	Multi-Stage or Multi-Compressor	More than two compressors, often used for large-capacity units or those requiring redundancy. Provides increased cooling power and failsafe operation. Energy Consumption: 20–30+ kWh/day.	[165]
3. Based on Form Factor	Upright ULT Freezers	Most common in labs. Easy access with adjustable shelves or racks. Capacity typically ranges from 300–800+ liters. Energy Consumption: 16–25 kWh/day. Examples: Thermo TSX Series, PHCbi MDF-DU702VH.	[4,8,83,98]
	Chest ULT Freezers	Top-opening design offers better insulation and temperature stability. Generally more energy-efficient than upright models. Capacity: 300–900 L. Energy Consumption: 12–18 kWh/day. Examples: So-Low U85-25, Stirling SU105UE.	[164]
	Under-Counter ULT Freezers	Compact and space-saving, often used in clinical or point-of-care labs. Capacity: 100–200 L. Energy Consumption: 6–10 kWh/day. Examples: PHCbi MDF-U33, Thermo Fisher TSX Series Compact.	[8]
	Portable/Mobile ULT Freezers	Designed for safe transport of biological samples at ultra-low temperatures. May be battery-powered, compressor-driven, or use dry ice. Energy Consumption: 2–6 kWh/day. Examples: Stirling Ultracold ULT25NEU.	[164]
4. Based on Application or Special Features	Energy-Efficient or Green ULT Freezers	Use hydrocarbon refrigerants (e.g., R-170, R-290) with low global warming potential (GWP). ENERGY STAR^®^ certified, optimized for low power consumption. Energy Consumption: 6–14 kWh/day. Examples: PHCbi VIP ECO Series, Thermo TSX Series.	[51,56,98]
	ULT Freezers with Redundant Compressor Systems	High-reliability systems featuring backup compressors or modular components for critical storage applications such as biobanking or clinical trials. Energy Consumption: 20–30+ kWh/day. Examples: Custom or pharma-grade configurations.	[165]

**Table 4:** Comparative analysis of low-temperature cooling systems.

Features	Hermetic Compressor	Free-Piston Engine	Multi-Compressors
References	[156,164,165,166]	[156,164,165,166]	[156,164,165,166]
Capacity	706 L	780 L	59,720 L
Temperature Range	−85 °C to −40 °C	−86 °C to −20 °C	−60 °C to 0 °C
Construction Material	AISI 304 stainless steel	Blank insulation panels	Special design with two systems
Cooling Technology	Hermetic compressors	Free-piston Stirling	Dual cooling system
Energy Consumption	Energy saving strategies	~6.67 kWh/d, up to 40% less energy	Requires maintenance for stable operation
Connectivity	USB, SIM, Wi-Fi, Ethernet	Remote monitoring	Limited options
Temperature Stability	Superior thermal performance	±1 °C	Constant temperature control
Operating Safety	Safety thermostats	Lock and PIN for access	Diagnostics and warnings
Maintenance Procedures	Regular programs necessary	Maintenance with GUI and automated monitoring	Regular maintenance and checks with automated diagnostics
Useful Life	10–12 years	12 years	10–12 years
Refrigerant Safety	HCFC or CFC-free R-170 and R-1270	Uses R-170 (Ethane), eco-friendly	Uses HFCs, requires caution due to flammability
Operating Noise	Noise during operation	<48 dB(A)	Noise during operation
Environmental Policy	Eco-friendly refrigerants	Uses natural refrigerants	Low ozone depletion potential
Resistance to Temperature Fluctuations	High	High	High via dual compressors
Reliability Price	High reliability	Variable operation with minimal maintenance	Excellent due to redundancy
Measurement	1990 × 1060 × 1000 mm	1994 × 870 × 915 mm	10,945 × 2154 × 2896 mm

**Table 5:** Regulatory requirements.

Source	Title of the Regulation	Article	References
EU	Eudralex; Volume 4 GMP Guidelines	Volume 4	[170]
	Good Distribution Practice of Active Substances for Medicinal Products for Human Use (2015/C 95/01)	2015/C 95/01	[171]
	Directive 2000/54/EC Protection of Workers from Risks Related to Exposure to Biologic Agents at Work	2000/54/EC Annex V and VI	[172]
	Directive 2009/41/EC on the Contained Use of Genetically modified Micro-organisms	2009/41/EC	[173]
	European Medicines Agency Scientific Guidance Documents on Biological Drug Substances	N/A	[174]
US FDA	Title 21 Code of Federal Regulations, Electronic Records, and Electronic Signatures	Part 11	[175]
	Title 21 Current Good Manufacturing Practice In Manufacturing, Processing, Packing, Or Holding Of Drugs; General	Part 210	[176]
	Title 21 Code of Federal Regulations, Current Good Manufacturing Practice for Finished Pharmaceuticals	Part 211	[176]
ASME	Bioprocessing Equipment	ASME BPE-2014	[159]
PIC/S	Guide to Good Manufacturing Practice for Medicinal Products, Part I	PE-009-9	[159]
	Guide to Good Manufacturing Practice for Medicinal Products, Part II	PE-009-11	[159]
ISPE	Baseline Guide: Biopharmaceuticals	Volume 6, 2nd	[21,22]
	Baseline Guide: Commissioning and Qualification	Volume 5, 2nd	[21,22]
	Good Practice Guide—Cold Chain Management	2011	[21,22]
	Good Practice Guide—Controlled Temperature Chamber Mapping and Monitoring	2016	[21,22]

## Data Availability

No new data were created or analyzed in this study. Data sharing is not applicable to this article.
